# Alternative ANKHD1 transcript promotes proliferation and inhibits migration in uterine corpus endometrial carcinoma

**DOI:** 10.1038/s41525-022-00321-0

**Published:** 2022-09-29

**Authors:** Wenhuizi Sun, Runzhi Huang, Zhenyu Li, Yaru Zhu, Yan Bai, Siyu Wu, Jingshuai Wang, Yan Xiao, Shuyuan Xian, Xiaowen Tong, Jie Zhang, Yi Guo, Yiqin Ouyang

**Affiliations:** 1 Department of Obstetrics and Gynecology, Tongji Hospital, School of Medicine, Tongji University, Shanghai, 200065 China; 2 School of Medicine, Tongji University, Shanghai, 200092 China; 3 Department of Burn Surgery, The First Affiliated Hospital of Naval Medical University, Shanghai, 200433 China; 4 Department of Urology, Tongji Hospital, School of Medicine, Tongji University, Shanghai, 200065 China; 5 Department of Pathology, Beijing Shijitan Hospital, Capital Medical University, Beijing, 100038 China; 6 Department of Obstetrics and Gynecology, Shanghai East Hospital, School of Medicine, Tongji University, Shanghai, 200120 China; 7 Department of Oncology, Tungwah Hospital of Sun Yat-sen University, Guangdong, 523110 China; 8 Key Laboratory of Spine and Spinal cord Injury Repair and Regeneration, Ministry of Education, School of Medicine, Tongji University, Shanghai, 200065 China

**Keywords:** Endometrial cancer, Gene regulation, Gene regulatory networks, Metastasis

## Abstract

Alternative splicing (AS) is common in gene expression, and abnormal splicing often results in several cancers. Overall survival-associated splicing events (OS-SEs) have been used to predict prognosis in cancer. The aim of this study was to investigate the presence and function of OS-SEs in uterine corpus endometrial carcinoma (UCEC). Based on TCGA and TCGASpliceSeq databases, gene expression and the AS data of UCEC samples were retrieved. An alternate terminator of ANKHD1 transcripts named ANKHD1-BP3 was found to be significantly related to metastasis and OS in UCEC and significantly associated with HSPB1. The upregulated expression of HSPB1 induced downregulation of ANKHD1-BP3 and promoted tumor metastasis. These findings indicate that HSPB1, a splicing factor, regulates the expression of ANKHD1-BP3 to promote metastasis in UCEC.

## Introduction

Alternative splicing (AS) is a common regulatory mechanism in gene expression, which allows multiple mRNA transcripts from a single gene. A total of 20,000 protein-coding genes may generate a larger number of variants than previously thought. The traditional classification of AS modes include exon skipping (ES), alternate promoters (AP), alternate terminators (AT), alternate acceptors (AA), alternate donors (AD), retained introns (RI), and exclusive exons (ME)^1,2^. AS is involved in human development, with 95% of genes processed by AS reported in a genome-wide study^2^. Abnormal splicing may lead to aberrant biological function and result in many diseases, including some cancers^3^. Some AS events (ASEs) associated with survival and prognosis in cancer patients are called overall-survival-associated splicing events (OS-SEs). These ASEs are regulated by splicing factors (SFs). Some recent studies reported that the mutational network between SFs and ASEs is associated with tumorigenesis and metastasis^4^, and could also be regarded as a predictor of prognosis in cancer patients^5,6^.

Uterine corpus endometrial carcinoma (UCEC) is one of the three most common cancers in women^7^. The American Cancer Society estimates that there will be 66,570 new cases of UCEC and 12,940 UCEC-related deaths in the USA in 2021^8^. Primary UCEC may be treated by surgery with or without radiation and hormone therapies. However, once UCEC metastasis occurs, therapeutic effects are limited^9,10^. Bone, lymph node, and omentum are the major metastasis sites in UCEC^11–15^, which leads to low quality of life and poor prognosis^11^. No effective biomarker is available for the prognosis or metastasis of UCEC. Moreover, there are no studies focusing on the interaction network between SFs and ASEs. Therefore, identifying the OS-SEs and exploring the potential mechanism underlying the SFs and ASEs might be a feasible method to predict the prognosis and metastasis of UCEC. In this study, we investigated the relationship between SFs and OS-SEs and the OS-SE biological effects in UCEC.

## Results

### Identification of ASEs and OS-SEs and construction of UpSet plots in UCEC

**Fig. 1:**
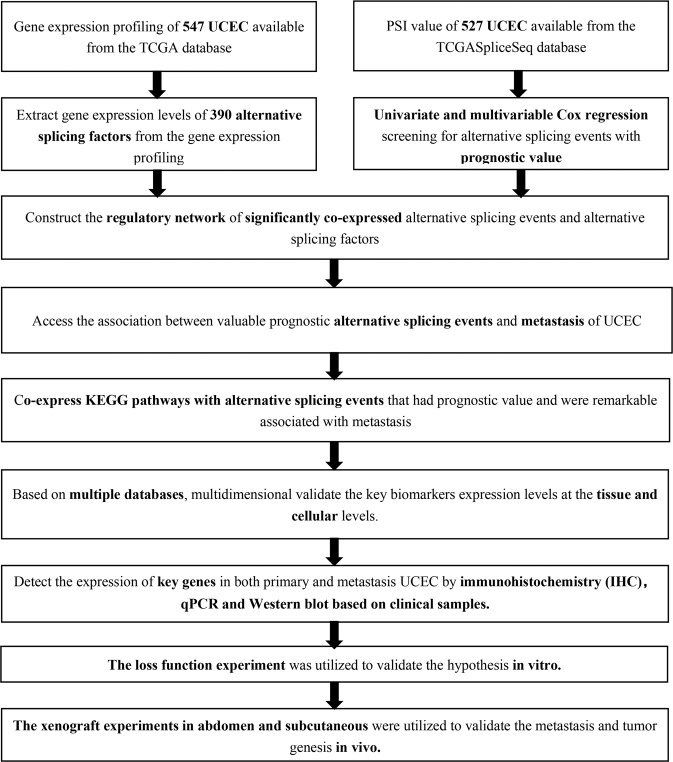
Analysis process presentation. The flow chart of the analysis process (a).

**Fig. 2:**
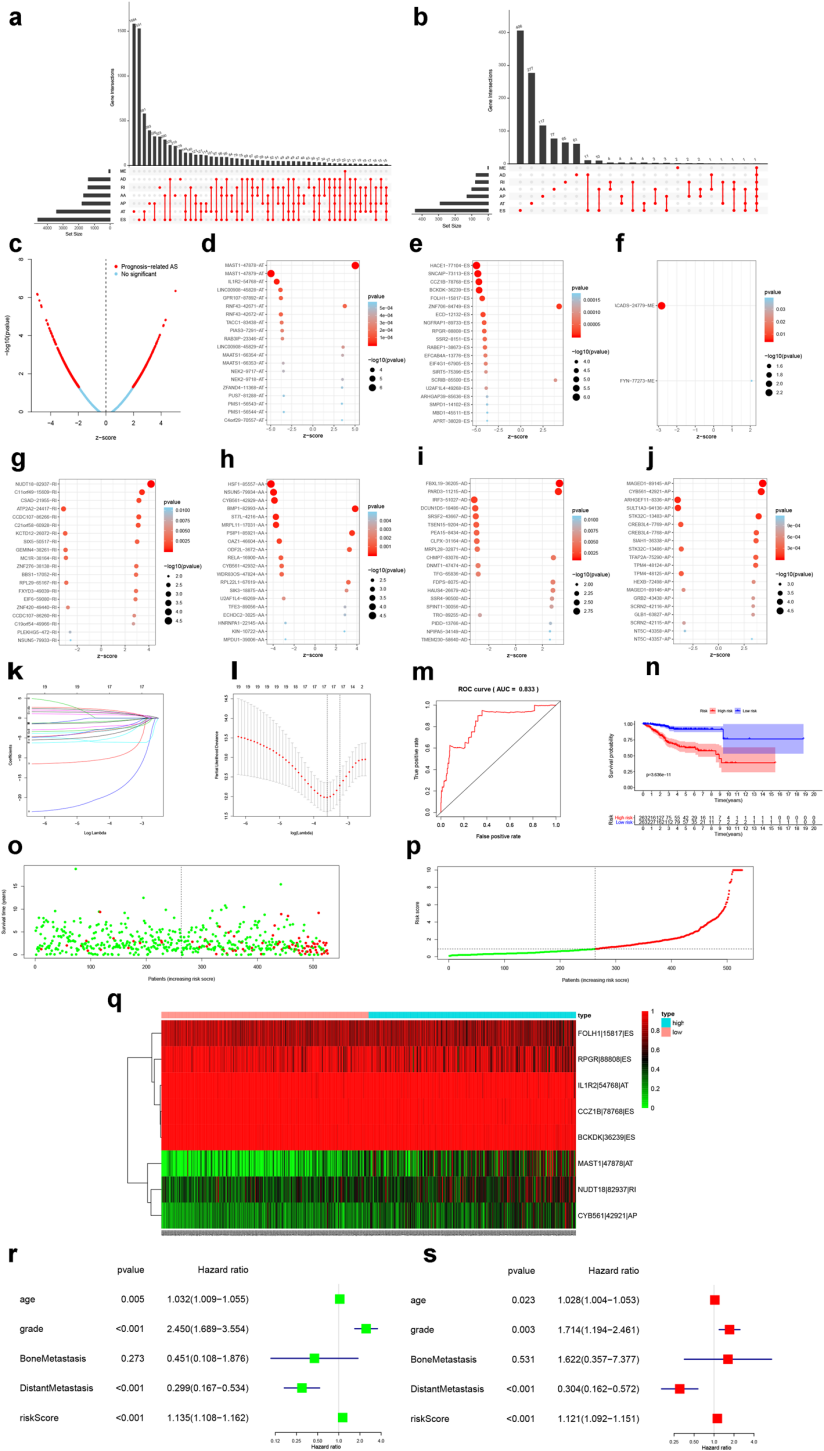
Identification of prognostic AS. UpSet plots of alternative splicing events (**a**) and alternative splicing events related to survival (**b**). Volcano plot of alternative splicing events to identify overall-survival-associated splicing events (**c**). Bubble plots of the top 20 overall-survival-associated splicing events (**d**–**j**). Lasso regression analyses of the top 20 overall-survival-associated splicing events (**k**, **l**). ROC curve (**m**), survival curve (**n**), scatterplot (**o**), and risk plot (**p**) of the risk model. The heatmap of the overall-survival-associated splicing events (**q**). Forest plots of univariate cox regression analysis (**r**) and multivariate cox regression analysis (**s**).

A flow chart shows the analysis of this study (Fig. 1). Based on the TCGA database, 547 primary UCEC samples were analyzed (Table 1). We combined the gene name, TCGASpliceSeq database AS ID of each ASE, and the splicing pattern. For example, “ANKHD1 | 73653|AT,” where ANKHD1 is the gene name, 73653 is the AS ID, and AT the splicing pattern. A total of 14,474 ASEs in 7965 genes were identified in patients with UCEC, including 1713 AAs in 1583 genes, 1413 ADs in 1282 genes, 1799 APs in 1690 genes, 3418 ATs in 3321 genes, 4600 ESs in 4537 genes, 109 MEs in 22 genes, and 1422 RIs in 1325 genes. Therefore, different splicing patterns were found in one gene. In addition, ASEs in all primary UCEC samples are shown in Fig. 2a, and ASEs related to overall survival are illustrated in Fig. 2b. In addition, ES was the most significant splicing pattern related to UCEC prognosis. Volcano plots of AS events was used to identify OS-SEs (Fig. 2c), and the seven bubble plots show the top 20 OS-SEs in each splicing pattern. MAST1 | 47878|AT, HACE1 | 77104|ES, ACADS | 24779|ME, NUDT18 | 82937|RI, HSF1 | 85557|AA, FBXL19 | 36205|AD, and MAGED1 | 89145|AP were the most significant in each splicing pattern (Fig. 2d–j).

**Table 1:** Baseline information of 547 patients diagnosed with uterine corpus endometrial carcinoma is available from the TCGA.

Variables	Total Patients (*N* = 547)
**Age, years**	
Mean ± SD	63.93 ± 11.14
Median (Range)	64 (31–90)
**Grade**	
G1	99 (18.10%)
G2	122 (22.30%)
G3	315 (57.59%)
High grade	11 (2.01%)
**Metastasis**	
Distant metastasis only	32 (88.89%)
Bone metastasis and distant metastasis	4 (11.11%)

### Construction of the multivariate model

The top 20 OS-SEs were processed using Lasso regression to avoid over-fitness in the multivariate model. The results show that FOLH1 | 15817|ES, RPGR | 88808|ES, IL1R2 | 54768|AT, CCZ1B | 78768|ES, BCKDK | 36239|ES, MAST1 | 47878|AT, NUDT18 | 82937|RI, and CYB561 | 42921|AP were integrated in the prediction model (Fig. 2k, l). ROC curves were utilized to access the accuracy of the prediction model, and the area under the curve (AUC) was 0.833 (Fig. 2m). Further, we set the risk score to 0.900 as the cutoff and divided patients into high- and low-risk groups according to their risk score. Kaplan–Meier curves were used to evaluate the prognostic accuracy of the risk score (*P* < 0.001) (Fig. 2m). Risk curves and scatterplots were applied to illustrate the risk score and clinical status, and red and green represent a high and low risk, respectively (Fig. 2o, p). In addition, the expression levels of each OS-SEs are illustrated in heatmaps. PSI values of FOLH1 | 15817|ES, RPGR | 88808|ES, IL1R2 | 54768|AT, CCZ1B | 78768|ES, and BCKDK | 36239|ES were significantly higher in the high-risk group, whereas MAST1 | 47878|AT, NUDT18 | 82937|RI, and CYB561 | 42921|AP were lower in the low-risk group (Fig. 2q).

### Independent prognostic analysis

To evaluate the risk score and other clinical characteristics in prognosis, univariate and multivariate Cox regression analyses were performed. The results of univariate (HR = 1.135, 95% CI (1.108–1.162), *P* < 0.001) and multivariate (HR = 1.121, 95% CI (1.092–1.151), *P* < 0.001) Cox regression analyses show that the risk score could be regarded as an independent prognostic factor in UCEC (Fig. 2r, s).

### Construction of a network between OS-SEs and SFs related to metastasis

**Fig. 3:**
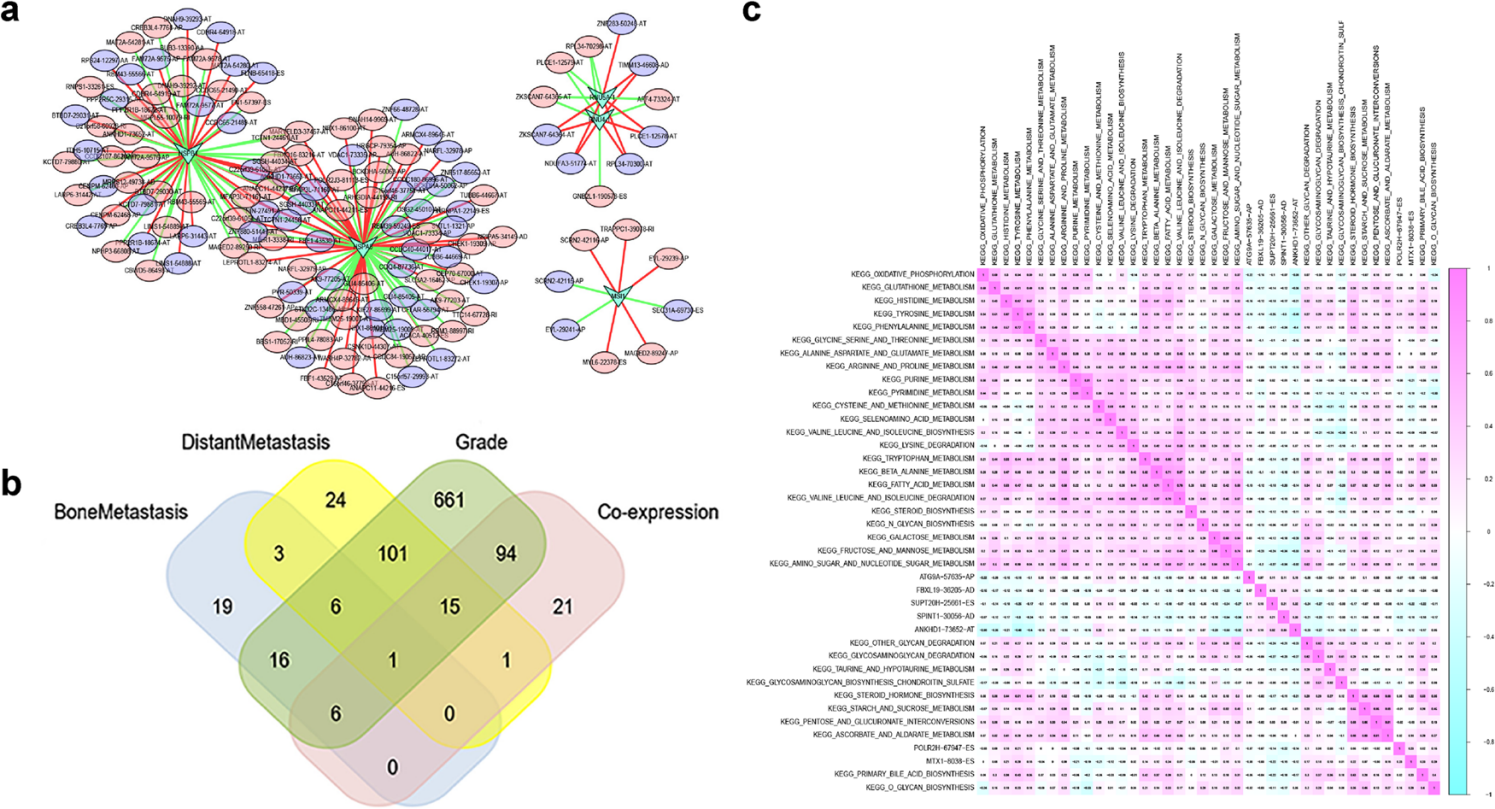
Construction of prognostic AS regulation network. The network constructed for splicing factors and overall-survival-associated splicing events (**a**). Venn plot showed the relationship between overall-survival-associated splicing events and bone metastasis/distant metastasis/grade/co-expression (**b**). Gene set variation analysis (GSVA) showed the co-expression of Kyoto Encyclopedia of Genes and Genomes (KEGG) pathways and overall-survival-associated splicing events related to metastasis (**c**).

We constructed a network to identify the regulatory relationship between OS-SEs and SFs. The results show that HSPB1, HSPA8, RNU5A-1, RNU4-1, and MSI1 were significantly present in the regulatory network of several genes. Ten high-risk OS-SEs were positively regulated by HSPA8 and negatively regulated by HSPB1: MARVELD3 | 37467|AT, TCTN1 | 24461|AT, FBXO16 | 83216|AT, SGSH | 44034|AT, ANAPC11 | 44217|ES, MFAP3L | 71161|AT, C22orf39 | 61054|AT, MIER1 | 3338|RI, MAGED2 | 89250|RI, and LEPROTL1 | 83274|AT. Seven low-risk OS-SEs were negatively regulated by HSPA8 and positively regulated by HSPB1: C22orf39 | 61055|AT, ANKHD1 | 73653|AT, MFAP3L | 71160|AT, SGSH | 44033|AT, NIN | 27491|AT, TCTN1 | 24458|AT, and ZNF880 | 51447|AT. Further, HSPA8 was positively and negatively correlated with VDAC1 | 73334|AP and VDAC1 | 73335|AP (*P* < 0.001, R = 0.526 and R = −0.526), C22orf39 | 61054|AT and C22orf39 | 61055|AT (*P* < 0.001, R = 0.556 and R = −0.624), respectively, whereas HSPB1 was positively and negatively correlated with ANKHD1 | 73653|AT and ANKHD1 | 73652|AT, respectively (*P* < 0.001, R = 0.316 and R = −0.424) (Fig. 3a). Venn diagrams were utilized to illustrate the relationship between OS-SEs and bone metastasis/distant metastasis/grade/co-expression (Fig. 3b). Moreover, ANKHD1 | 73652|AT was related to bone metastasis (*P* = 0.035), distant metastasis (*P* = 0.011), OS (*P* < 0.001), and co-expression with HSPB1, FBXL19 | 36205|AD (bone metastasis: *P* = 0.007, distant metastasis: *P* = 0.004, OS: *P* < 0.001), POLR2H | 67947|ES (bone metastasis: *P* = 0.011, distant metastasis: *P* = 0.009, OS: *P* < 0.001), SPINT1 | 30056|AD ((bone metastasis: *P* = 0.024, distant metastasis: *P* < 0.001, OS: *P* < 0.001), MTX1 | 8038|ES (bone metastasis: *P* = 0.034, distant metastasis: *P* < 0.001, OS: *P* < 0.001), ATG9A | 57635|AP (bone metastasis: *P* = 0.039, distant metastasis: *P* = 0.005, OS: *P* < 0.001), and SUPT20H | 25661|ES (bone metastasis: *P* = 0.041, distant metastasis: *P* = 0.008, OS: *P* < 0.001) were also significantly related to bone metastasis, distant metastasis, and OS (Supplementary Fig. 1a–u). Furthermore, ANKHD1 | 73652|AT was defined as ANKHD1-BP3 using the NCBI gene database (https://www.ncbi.nlm.nih.gov/gene).

### Comprehensive analysis of OS-SEs related to metastasis and survival-related pathways

We applied GSVA and univariate Cox analysis and found 33 KEGG pathways related to OS. Moreover, we utilized Pearson analysis to co-express the prognostic KEGG pathways and ASEs related to metastasis and OS (Fig. 3c). The results show that AT of ANKHD1-BP3 was significantly related to HSPB1 (*P* < 0.001, R = 0.316 and −0.424) and tyrosine metabolism (*P* < 0.001, R = −0.46). In addition, oxidative phosphorylation (*P* < 0.001, R = −0.36), glutathione metabolism (*P* < 0.001, R = −0.35), phenylamine metabolism (*P* < 0.001, R = −0.40), and fructose and mannose metabolism (*P* < 0.001, R = −0.33) were the other four top pathways.

### Multidimensional validation

Multiple online databases were applied to validate the levels of gene expression at the cellular and tissue levels. In addition, EGFR, SRC, TH, TYK2, and TAT were the top five genes related to the tyrosine metabolism pathway in the Genecard database. Therefore, we selected HSPB1, ANKHD1, EGFR, SRC, TH, and TYK2 for validation (Supplementary Fig. 2). HSPB1, EGFR, and TYK2 were highly expressed in tumor tissue in the Human Protein Atlas database. HSPB1 was highly expressed in normal tissue in the GTEx database (Supplementary Fig. 3). HSPB1, EGFR, and TYK2 were highly expressed, but ANKHD1, TH, and TAT were expressed at low levels in UCEC tissues in the PROGgeneV2 database (Supplementary Fig. 4). HSPB1, ANKHD1, EGFR, SRC, and TYK2 were highly expressed both in UCEC and in normal tissues in the GEPIA database (Supplementary Fig. 5). HSPB1, ANKHD1, EGFR, SRC, TH, and TYK2 were highly expressed in UCEC tissues in the UCSC xena database (Supplementary Fig. 6). HSPB1, ANKHD1, EGFR, and TYK2 were highly expressed in UCEC tissues in the SurvExpress database (Supplementary Fig. 7). HSPB1, ANKHD1, EGFR, SRC, and TYK2 were highly expressed, whereas HSPB1, SRC, and TYK2 were expressed at higher levels in UCEC than in normal tissue in the UALCAN database (Supplementary Fig. 8a–n). The overall-survival curves of HSPB1, ANKHD1, EGFR, SRC, TH, TYK2, and TAT were shown in Supplementary Fig. 8o–u. HSPB1, EGFR, SRC, and TH were highly expressed, but TAT had low expressed levels, in UCEC tissues in the Linkedomics database (Supplementary Fig. 9). HSPB1, ANKHD1, EGFR, and TYK2 were highly expressed in UCEC tissues in the cBioportal database, and the relationship evaluated by the cBioportal database was significant (Supplementary Fig. 10) (Supplementary Table 1). HSPB1 was highly expressed, but ANKHD1 and TH were expressed at low levels in UCEC tissues in the Expression atlas. HSPB1, EGFR, and TYK2 were highly expressed, but ANKHD1 was expressed at low levels in UCEC tissues in the Oncomine database (Supplementary Fig. 11). Finally, HSPB1, EGFR, SRC, and TYK2 were highly expressed, but TH and TAT were expressed at low levels, in UCEC at the cellular level in the CCLE database (Supplementary Fig. 12). Using these data, we constructed a protein-protein interaction network using the String database (Supplementary Fig. 13a, b). Overall, HSPB1, EGFR, SRC, and TYK2 were highly expressed in UCEC, whereas TH and TAT were expressed at low levels. All results of validation were summarized in Supplementary Table 2.

**Fig. 4:**
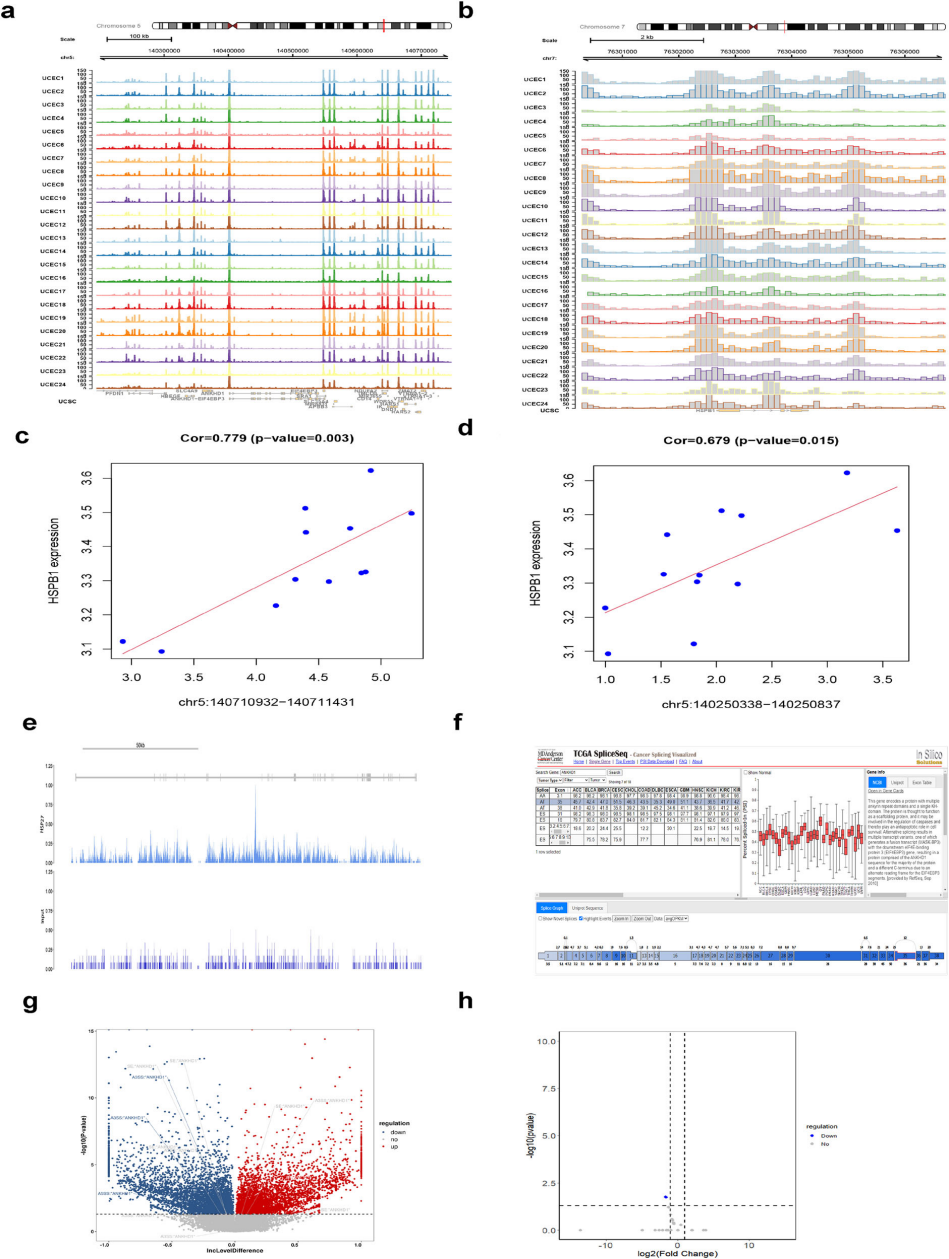
Direct AS mechanism validation of ANKHD1. ATAC-seq of HSPB1 (**a**) and ANKHD1 (**b**). The correlation of HSPB1 and the chromatin accessibility of ANKHD1 (**c**, **d**). Chip-seq of HSPB1 (**e**) in HEC-1b cells. The splicing pattern plot (**f**). Overexpression of HSPB1 generated ASEs (**g**) and ANKHD1-BP3 (**h**).

To further explore the regulatory relationship between HSPB1 and ANKHD1, we applied direct mechanism validations. ATAC-seq of HSPB1 (Fig. 4a) and ANKHD1 (Fig. 4b) was performed, and the results show open domains of chromatin. Moreover, a correlation between HSPB1 expression and chromatin accessibility was found (Fig. 4c, d). In addition, the Chip-seq of HSPB1 (Fig. 4e) in HEC-1b cells was utilized, and peaks in the plot show that HSPB1 and ANKHD1 can bind to each other’s genes. A schematic diagram of ANKHD1 (Fig. 4f) illustrates the splicing pattern analyzed by TCGASpliceSeq data.

RNA sequencing showed that 37,679 ASEs were generated after overexpression of HSPB1, among which 15,696 were upregulated, 3791 were unchanged, and 18,191 were downregulated. There were 4195 upregulated SEs and 5112 downregulated ASEs with significant significance. The top five shear modes were “A3SS” with 3518, “A5SS” with 2259, “MXE” with 3396, “RI” with 2541, and “SE” with 25,964. Among them, there were 865 significant “A3SS,”647 “A5SS,” 825 “MXE,”491 “RI,” and 6479 “SE” (Fig. 4g). Further analysis of the associated splicing variants of ANKHD1 resulted in 24 ASEs. There were only two statistically significant ASEs (*p* < 0.05), which were associated with the splicing variant ANKHD1-BP3 (Fig. 4h).

### Validation using clinical samples

**Fig. 5:**
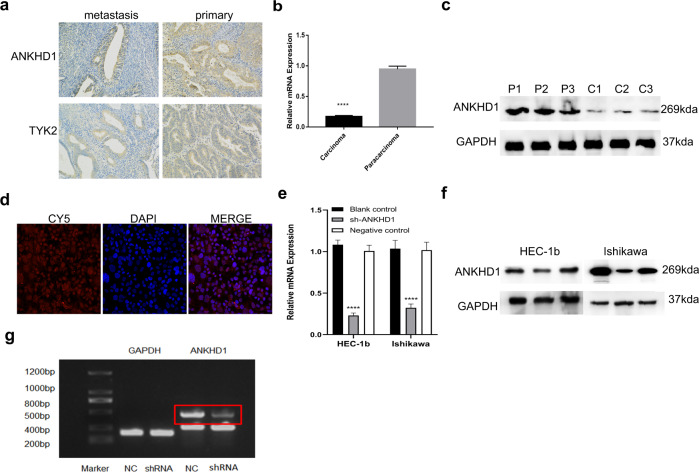
The expression and location of ANKHD1 in clinical samples and UCEC cells. Immunohistochemistry of ANKHD1 (**a**) between metastasis and primary UCEC samples. RT-PCR (**b**) and Western blot (**c**) for the expression of ANKHD1 in carcinoma and para-carcinoma. Immunofluorescence staining of ANKHD1 (**d**). RT-PCR (**e**) and Western blot (**f**) of blank, shRNA, and NC group. The result of DNA electrophoresis (**g**). ****p < 0.0001. Data shown are the mean ± SD of at least three independent experiments.

The associationship between ANKHD1 expression and tumor metastasis, ANKHD1 protein levels were detected by immunohistochemistry (IHC) (Fig. 5a). ANKHD1 was present in both primary and metastatic UCEC samples, and the protein expression level in primary tissues was higher than that in metastatic tissues. RT-PCR (Fig. 5b) and WB (Fig. 5c) were used to detect the expression of ANKHD1 in carcinoma and para-carcinoma tissues. The results showed that the ANKHD1 level were higher in pericarcinomatous tissues than in cancer tissues. For further exploration, the location of ANKHD1 in cells was observed with immunofluorescence staining (Fig. 5d). The results showed that it was mostly present in the nucleus.

### Validation using the loss of function experiments in vitro and in vivo

**Fig. 6:**
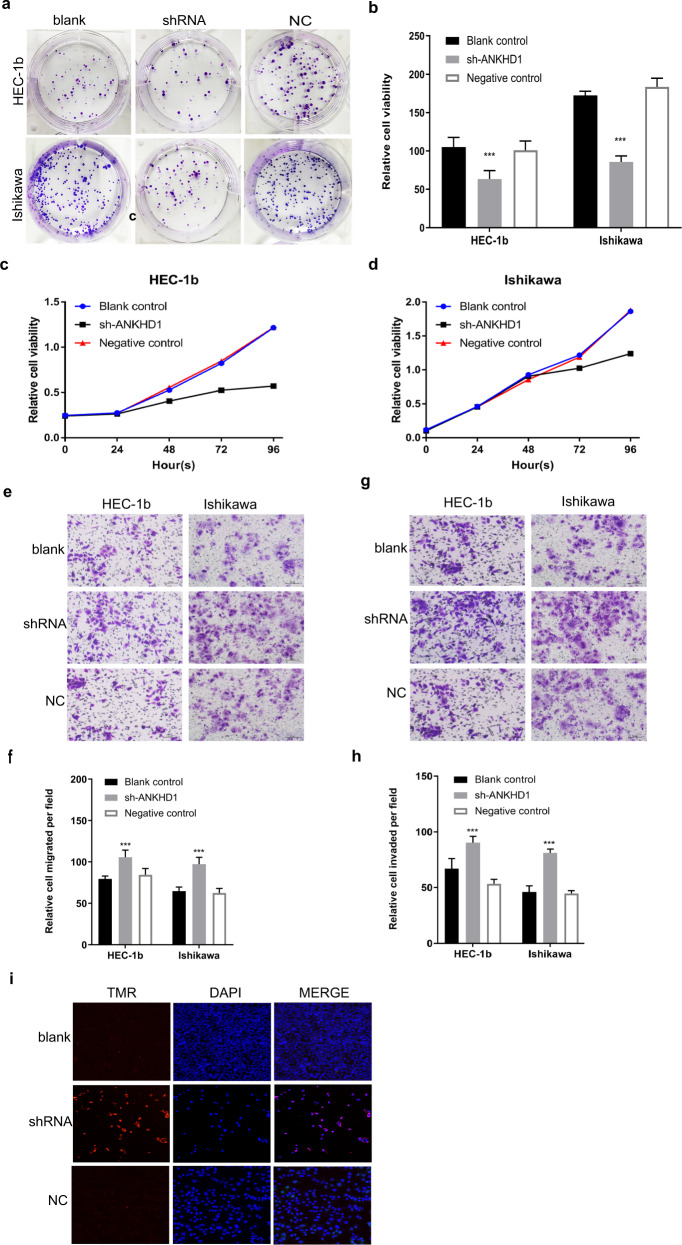
Loss of function experiment in vitro. The plate clone formation assay (**a**, **b**), CCK-8 assay (**c**, **d**), migration assay (**e**, **f**), invasion assay (**g**, **h**), and Tunel results (**i**) of blank, shRNA, and NC group. ***p < 0.001. Data shown are the mean ± SD of at least three independent experiments. Data shown are the mean ± SD of at least three independent experiments.

To further test the function of ANKHD1-BP3, RNA interference was used to deplete endogenous ANKHD1-BP3 in UCEC cells. RNA and protein levels in the blank, shRNA-ANKHD1-BP3, and NC groups were detected using qPCR (Fig. 5e) and WB (Fig. 5f) to determine the effects of ANKHD1-BP3 knockdown. The gene was successfully knocked down in 70% of cells. To further determine the specificity of shRNA-ANKHD1-BP3, DNA from shRNA-ANKHD1-BP3 cells was verified using electrophoresis (Fig. 5g), and gel DNA sequencing was performed (Supplementary Table 3). The expression of Ishikawa shRNA-ANKHD1-BP3 on agarose gel was lower than that of the NC group, and the sequencing results showed that it was a transcription of ANKHD1-BP3. The proliferation ability of UCEC cells was then tested in colony formation (Fig. 6a, b) and CCK-8 (Fig. 6c, d) assays. The results show that proliferation was weaker when ANKHD1-BP3 was knocked down in the shRNA-ANKHD1-BP3 group compared with that in the NC group. In contrast, migration (Fig. 6e, f) and invasion (Fig. 6g, h) abilities were enhanced in the shRNA-ANKHD1-BP3 group compared to those in the other groups. In addition, TUNEL assay results showed that apoptosis was increased in cells with ANKHD1-BP3 knocked down (Fig. 6i). We conclude that ANKHD1-BP3 promoted proliferation but inhibited metastasis, invasion, apoptosis, and necrosis of cells in vitro.

**Fig. 7:**
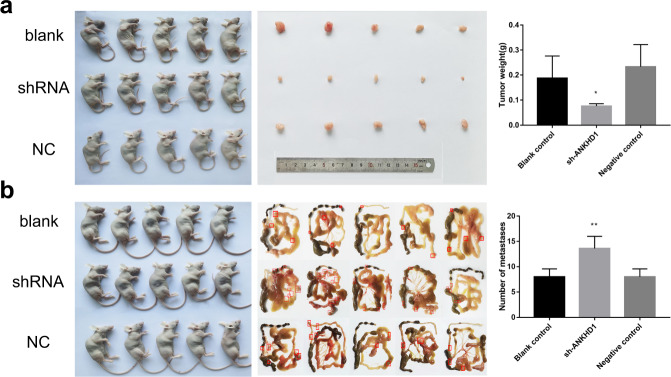
Loss of function experiment in vivo. The nude mice xenograft experiment in subcutaneous (**a**) and the peritoneal metastasis experiment (**b**). *p < 0.05; **p < 0.01. The data shown are the mean ± SD.

The effects of ANKHD1-BP3 on the growth of UCEC cells were also determined in vivo using a nude mouse xenograft UCEC model. Compared with the negative control, a smaller neoplasm formed in the nude mice injected with Ishikawa shRNA-ANKHD1-BP3 cells than in the subcutaneous injection group (Fig. 7a). Nevertheless, cells injected into the abdominal region spread to several organs. Additionally, more metastasis was observed in the Ishikawa shRNA subgroup than in the Ishikawa and Ishikawa NC subgroups (*P* < 0.05) (Fig. 7b). In summary, ANKHD1-BP3 promoted cell proliferation while inhibiting metastasis in vivo.

**Fig. 8:**
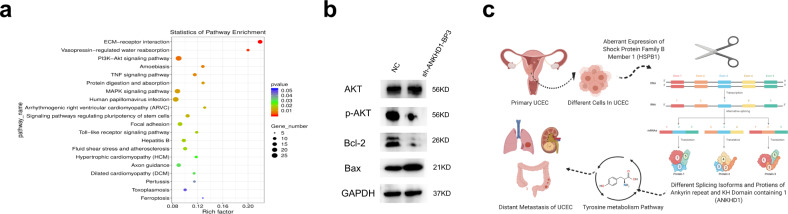
ANKHD1-BP3 regulates the activity of the PI3K-AKT pathway and the plot of the hypothesis. Bubble maps (**a**) of the KEGG pathway. ANKHD1-BP3 regulates the activity of the PI3K-AKT pathway in Ishikawa (**b**). The plot of hypothesis (**c**) showed that HSPB1 regulated ANKHD1, and the transcript of ANKHD1 was involved in the tyrosine metabolism pathway, which affected the distant metastasis. Figure 8c was created with BioRender.com.

To elucidate the mechanism underlying the ANKHD1-BP3 of UCEC cells, downstream molecules involved with ANKHD1 in Ishikawa were investigated. KEGG enrichment analysis was performed for differentially expressed genes in RNA sequencing data to obtain the mechanism pathways, and the top 20 KEGG pathways with the lowest *P* value were selected to draw bubble maps (Fig. 8a). The PI3K/AKT pathway was further verified in Ishikawa cells (Fig. 8b). When we knocked down ANKHD1-BP3, the expression of AKT1 remained unchanged, but the expression of p-AKT1 and Bcl-2 decreased, and the expression of Bax increased. The mechanism is shown in Fig. 8c.

## Discussion

In this study, we downloaded the gene expression data of 547 primary UCEC samples from the TCGA database and extracted 390 SFs. In addition, 527 PSI values of ASEs in UCEC were downloaded from the TCGASpliceSeq database. Using univariate Cox regression analysis to identify OS-SEs, FOLH1 | 15817|ES, RPGR | 88808|ES, IL1R2 | 54768|AT, CCZ1B | 78768|ES, BCKDK | 36239|ES, MAST1 | 47878|AT, NUDT18 | 82937|RI, and CYB561 | 42921|AP were found to be significantly related to UCEC by Lasso regression screening. In addition, we constructed a Cox model with OS-SEs selected by Lasso regression to predict UCEC prognosis, and multiple analyses show that the risk score was an independent prognosis factor. We co-expressed the SF and OS-SEs and then constructed an interaction network. Furthermore, we found that VDAC1 | 73334|AP and VDAC1 | 73335|AP, C22orf39 | 61054|AT and C22orf39 | 61055|AT, and ANKHD1 | 73653|AT and ANKHD1 | 73652|AT were significantly associated. OS-SEs associated with metastasis and OS were also identified, and ANKHD1 | 73652|AT was found to be significantly associated with both. OS-related KEGG pathways were selected by GSVA, and KEGG pathways and OS-SEs were co-expressed to identify the most significant pathways related to ANKHD1-BP3. The results show that tyrosine metabolism might be the potential downstream pathway for the regulatory interaction between HSPB1 (SF) and ANKHD1-BP3. ANKHD1-BP3 was highly expressed in primary UCEC as shown by IHC and was highly expressed in pericarcinomatous tissues as detected by RT-PCR and WB. In addition, ANKHD1-BP3 was mainly located in the nucleus in clinical samples, and ANKHD1-BP3 inhibited apoptosis and necrosis in vitro. Similarly, ANKHD1-BP3 promoted proliferation while inhibiting metastasis in vivo and in vitro. Sequencing showed that overexpression of HSPB1 resulted in 9308 ASEs, two of which were statistically significant and related to ANKHD1-BP3. Namely, the downregulation of ANKHD1-BP3 was related to HSPB1, which is consistent with our transcriptome sequencing results and with previous bioinformatics analysis.

HSPB1, also called HSP27, is a member of the heat shock protein family B and is up-regulated in cancer and oxidative stress^16^. It is highly expressed in some cancers, and its overexpression also leads to poor prognosis^17^, bone metastasis, and invasion^18^. ANKHD1, also called MASK, contains ankyrin repeat and KH domain and is involved in receptor tyrosine kinase signaling^19^. HSPB1 regulates β-catenin/SLUG pathway, which plays an important role in epithelial-to-mesenchymal transition (EMT)^20^. Similarly, decreased ANKHD1 expression reduces the SMYD-dependent activation SLUG expression, thus, affecting hepatocellular metastasis^21^. HSPB1 also inactivates the Hippo tumor-suppressor pathway and decreases the phosphorylation of YAP^22^. Downregulation of ANKHD1 suppresses the EMT by inactivating YAP1^23^. Additionally, HSP27 participates in the TGF-β/smad/p38 MAPK pathway in EMT^24^. Knockdown of ANKHD1 reduces matrix metalloproteinases (MMPs), and MMP-mediated activation of TGF-β results in EMT^23^. ANKHD1-BP3 is also associated with SH2, a nonreceptor protein-tyrosine phosphatase, to regulate cell proliferation, and SH2 is related to the MAPK pathway^25^. Therefore, we propose that the interaction between HSPB1 and ANKHD1 might affect EMT through the SLUG, Hippo, TGF-β, and MAPK pathways during UCEC metastasis.

Transcriptome sequencing analysis showed that ANKHD1-BP3 was the mainly expressed transcript in endometrial cancer cells, and the expression of this transcript was reduced when HSPB1 was overexpressed. Knockdown of this transcript inhibited the metastasis of endometrial cancer cells, which is inconsistent with previous studies. We suspect that different splice variants play different roles, and there might be transcripts of this gene with opposite effects on metastasis. For example, Liu et al. mainly focused on transcript 2 that did not contain the KH domain^26^. We focused instead on a transcript of the fusion gene ANKHD1-BP3. The function of this transcript has not yet been studied.

Because the tyrosine metabolism calculated by the bioinformatic analysis was a gene set instead of a specific pathway, we identified the top five genes, namely, EGFR, SRC, TH, TYK2, and TAT, which are tyrosine kinase receptors. Notably, the PI3K/AKT signaling pathway is one of the most common pathways downstream of the receptor tyrosine kinases^26^ and is involved in the regulation of numerous cellular activities, including cell growth, migration, differentiation, apoptosis, and energy metabolism^27–30^ AKT is activated by the PI3K/AKT pathway and regulates tumor progression. EGFR is an epidermal growth factor receptor, and the protein coded by EGFR is a member of the protein kinase superfamily. In a retrospective analysis, EGFR showed a clinical relationship with metastasis in UCEC^31^, where EGFR interacts with MUC13 to activate the EGFR/PI3K/AKT signaling pathway^32^. SRC is a nonreceptor tyrosine kinase that regulates the endothelial barrier to improve EMT invasion and metastasis^33^. Activation of SRC can promote fibroblast activation by activating the downstream PI3K/AKT and mTOR/p70S6K signaling pathways^34^. TYK2 is tyrosine kinase 2, and it activates the Stat3 pathway to promote liver cell invasion^35^.

The restricted size of our data and selection bias are the limitations of our analysis, and thus, we applied online multidimensional validation at the cellular and tissue levels to minimize these limitations. In terms of downstream mechanism, we only enriched related pathways from differential genes of RNAseq and only verified the relationship between UCEC in the PI3K/AKT pathway and apoptosis by WB, which was a superficial exploration at the level of mechanism. Moreover, for multidimension validation, only ANKHD1 was accessible. Thus, IHC and WB were used for validation. We emphasize that we performed a bioinformatic analysis and not a mechanistic study.

The expression levels of ANKDH1-BP3 were low in metastasis and were downregulated by HSPB1. The low level of ANKHD1-BP3 may affect the proliferation and apoptosis of UCEC cells by upregulating AKT phosphorylation and activating the PI3K/AKT signaling pathway.

We propose that HSPB1 negatively regulates ANKHD1-BP3 and that a low level of ANKHD1-BP3 promotes cell proliferation and affects metastasis via the PI3K/AKT signaling pathway in UCEC cell lines. Moreover, ANKHD1-BP3 may be used as a prognostic biomarker in UCEC.

## Methods

### Data extraction and UpSet plot construction

Clinical information data and quantified gene expression transcriptome profiling of 390 primary UCEC samples were downloaded from the TCGA database (https://tcgadata.nci.nih.gov/tcga/), including 404 alternative SFs. In addition, missing percent spliced in (PSI) values of less than 25% were gathered from 527 primary UCEC samples in the TCGASpliceSeq database (https://bioinformatics.mdanderson.org/TCGASpliceSeq/)^36^. The AS patterns included AA, AD, AP, AT, ES, ME, and RI^37^. Each ASE is described with the gene name, TCGASpliceSeq database AS ID, and splicing pattern. An UpSet plot is shown to summarize the ASE profile in UCEC.

### Construction UpSet plots of OS-SEs

Using the K-nearest neighbor algorithm, we inserted the missing expressed data in ASEs data. Data were filtered using three criteria, a mean PSI of less than 0.05, a standard derivation of less than 0.01 among all samples, and samples without follow-up recorded. Furthermore, ASEs combined with clinical information were analyzed with univariate Cox regression, and each prognostic value of ASEs was determined, and the OS-SEs were identified. UpSet plots were then set up to display OS-SEs. Afterward, *z*-score and −log10 (*P* value) were set as x-axis and y-axis, and volcano plots were used to illustrate the relationship between ASEs and prognosis. Finally, bubble plots were generated to display the top 20 significant OS-SEs in AA, AD, AP, AT, ES, ME, and RI.

#### Lasso regression and construction of a multivariate model with OS-SEs

The top 20 significant OS-SEs were integrated using Lasso regression to avoid the over-fitting of the multivariable model. Furthermore, a multivariate Cox regression model was set up based on the OS-SEs processed by Lasso regression, and a receiver operator characteristic (ROC) curve was applied to access its accuracy. We calculated the risk score using the following formula:where *n* represents the number of OS-SEs selected by Lasso regression, and β each regression coefficient of OS-SEs. Afterward, samples were divided into high- and low-risk groups according to the median risk score. Kaplan–Meier curves were utilized to evaluate the relationship between risk score and survival probability. In addition, the PSI values of ASEs between high- and low-risk groups in the final model were illustrated using scatterplots and risk curves, respectively.

#### Independent prognostic analysis

Univariate and multivariate Cox analyses were applied to determine the independent prognostic value of the multivariate model risk score. Baseline information, including age, grade, and metastasis were integrated into the multivariate Cox analysis.

#### Co-expression and construction of a network between SF and OS-SEs

Based on the SpliceAid2 database, SF data were downloaded^38^. To determine the relationship between SF expression and PSI values, SFs and prognostic OS-SEs were co-expressed, and Pearson correlation was utilized to analyze the 390 SFs and prognostic OS-SEs based on expression levels. We filtered the regulation relationship by discarding interactions with *P* < 0.001 and an absolute value of correlation coefficient >0.400. A network between SF and OS-SEs was developed using Cytoscape (3.7.1)^39^. In our network, ellipses and arrows show OS-SEs and SF, in which the red and blue ellipses represent a high and low risk of OS-SEs, respectively, whereas red and green lines link SFs and OS-SEs represented positive and negative regulation, respectively.

#### Identification of OS-SEs related to metastasis

We applied the Kruskal–Wallis test and Mann–Whitney–Wilcoxon test to identify OS-SEs related to metastasis. In addition, according to the network analysis, these ASEs were also related to the regulatory network of SFs.

#### Co-expression analysis between ASEs and KEGG pathways

The prognostic signaling Kyoto Encyclopedia of Genes and Genomes (KEGG) pathways estimated by gene set variation analysis (GSVA) were identified through univariate Cox analysis. A co-expression analysis between the prognostic pathways and metastasis-specific OS-SEs were conducted to identify potential downstream pathways.

### Multidimensional validation

To minimize selection bias and the effects of limited data size, several databases were used to validate the expression of biomarkers we accessed at the cellular and tissue levels. Firstly, we used Genecard (https://www.genecards.org/) to find the top five genes related to pathways that we identified. In the top five genes, significant SF and OS-SEs were incorporated for further validation. Moreover, the Human Protein Atlas^40^, Genotype-Tissue Expression (GTEx)^41^, PROGgene Version2^42^, Gene Expression Profiling Interactive Analysis (GEPIA)^43^, UCSC xena^44^, SurvExpress^45^, Ualcan^46^, Linkedomics^47^, cBioportal^48^, Expression atlas^49^, and Oncomine^50^ databases were utilized to validate the results in multiple dimensions. Furthermore, the Cancer Cell Line Encyclopedia (CCLE)^51^ was applied to validate the data at the cellular level. The String database^52^ was applied to construct a gene regulatory network at the molecular level.

To further explore the mechanism of AS, the Assay for Targeting Accessible Chromatin with high throughput sequencing (ATAC-seq) was utilized to validate chromatin accessibility^53^. Chromatin Immunoprecipitation sequencing (Chip-seq) was also applied to validate the binding domain of ASE and SF. However, the anti-HSPB1 antibody was absent, and thus, HSPB90B1 was applied to replace HSPB1 because HSPB1 is homologous to HSPB90B1 (GSE126151)^54^. The Cistrome data browser^55^ was utilized.

### Patients and specimens

A total of 36 tumor specimens, 15 metastatic UCEC tissues, 15 primary UCEC tissues, and 6 additional UCEC tissues with paired non-tumor tissues were collected between January 2019 and January 2020 at the Tongji Hospital affiliated with the Tongji University School of Medicine. Written informed consent was obtained from all patients, and the procedures were approved by the Institutional Research Ethics Committee of Tongji Hospital, affiliated with Tongji University School of Medicine. Immunohistochemistry was used for the 15 primary and 15 metastatic primary UCEC tissues, reverse-transcription polymerase chain reaction (RT-PCR) was performed on 6 UCEC tissues and paired pericancerous tissues, and western blotting was performed on three of the six patient tumor tissues and paired pericancerous tissues.

### Immunohistochemistry

Tissue blocks were cut into 4-µm-thick sections, deparaffinized, rehydrated, and stained overnight at 4 °C using an Ultrasensitive TM S-P system (KIT-9710; MaiXin, Fujian, China), and incubated with antibodies against ANKHD1 (1:100, cat. no. ab199164; Abcam, Cambridge, UK). Tissue sections were incubated with a secondary antibody labeled with biotin at 37 °C for 30 min (Ultrasensitive TM S-P, MaiXin). Diaminobenzidine tetrahydrochloride substrate (MaiXin) was used as the chromogen. The number of cells expressing ANKHD1 was classified into five grades: 0 points (no cell staining), 1 point (1–25% cell staining), 2 points (26–50% cell staining), 3 points (51–75% cell staining), and 4 points (76% + cell staining). Based on the intensity of cell staining, ANKHD1 expression was classified into four grades: 0 (no staining), 1 (light yellow particles), 2 (yellow particles), and 3 (dark yellow or tan particles). The final score of each section was the number of cells in the section multiplied by the staining score. A score less than 2 was considered negative, whereas a score greater than or equal to 2 was considered positive. Phosphate-buffered saline (PBS) and goat serum were used as negative controls.

### Reverse-transcription quantitative PCR and agarose gel electrophoresis

Total tissue RNA was extracted using the RNA Fast 200 kit (Fastagen, Shanghai, China) and reverse-transcribed using the TB Green Premix Ex TaqTM Kit (Takara, Kyoto, Japan) according to the manufacturer's instructions. RNA quality check was performed using a spectrophotometer (acceptable A260/280 ratio between 1.8 and 2.0). cDNA was used as a template for RT-PCR at 95 °C for 5 min, denaturation for 5 s at 95 °C, and annealing at 60 °C for 30 s (40 cycles). The primer sequences were as follows: ANKHD1-BP3, forward CCAGATCCTGCTTGGAACCC, reverse TGTTTCCAATATGAGGTGCCCA; HSPB1, forward GCTTCACGCGGAAATACACG, reverse GTGATCTCGTTGGACTGCGT; and GAPDH, forward GGAGCGAGATCCCTCCAAAAT, reverse GGCTGTTGTCATACTTCTCATGG. qPCR reactions were performed in triplicate and the comparative CT method (2 − ΔΔCT method) was used to calculate the relative gene expression levels.

A 1% agarose gel was prepared using agarose powder (Biowest, Madrid, Spain) and Tris-acetic acid (TAE) (Beyotime, Shanghai, China), and the DNA amplification products were mixed with loading buffer (Beyotime). Samples were then loaded for electrophoresis. After electrophoresis, the gel was placed into a TAE solution (Beyotime) containing 0.5 μg/mL ethyl bromide for 30 min of staining. The bands were observed using a gel imager (Biorad, Hercules, CA, USA). The gel containing the target bands was then cut for DNA sequencing (Genewiz, Suzhou, China).

### Western blotting

Western blot (WB) analysis was performed to evaluate ANKHD1 expression in both tissues and cells before and after transfection. Proteins were extracted from cells and the protein concentration was determined using a bicinchoninic acid (BCA) kit (Beyotime). After blocking, membranes (Millipore, Boston, MA,USA) were incubated with anti-ANKHD1 antibody (ab117788, Abcam; dilution 1:2000), anti-AKT antibody(AF1777, Biotime; dilution 1:1000), anti p-AKT antibody(AF1546, Biyotime; dilution 1:1000), anti-BAX antibody(ab32503, Abcam; dilution 1:2000), anti-Bcl-2 antibody(ab182858, Abcam; dilution 1:2000) and then incubated overnight at 4 °C. Subsequently, horseradish peroxidase-conjugated goat anti-rabbit immunoglobulin G (ab205718, Abcam; dilution 1:5000) was added to the membranes and incubated for 1 h before detection. All blots derive from the same experiment and were processed in parallel.

### Immunofluorescence staining

Cells were fixed for 30 min at 25 °C in 4% paraformaldehyde in PBS, permeabilized with Triton X-100 (Sangon Biotech, Shanghai, China), and then blocked with 1% bovine serum albumin for 1 h at 25 °C. Cells were incubated with antibody against ANKHD1 (BS-5831R, Bioss, Beijing, China; dilution 1:100). After washing three times (5 min per wash) with PBS, the cells were incubated with fluorescein isothiocyanate-conjugated antibody (ab150077, Abcam; dilution 1:500). Nuclei were counterstained at 25 °C for 10 min with DAPI (Beyotime).

### Cell culture and transfection

Human EC cell lines Ishikawa(FH0305, FuHeng Biology, China) and HEC-1b(GDC0129, CDCC, China) were maintained in 89% DMEM media (GIBCO, Carlsbad, CA, USA) supplemented with 10% FBS (GIBCO) and 100 U/mL penicillin (Hyclone, Logan, UT, USA) in a humidified atmosphere containing 5% CO_2_ at 37 °C. A lentivirus transfection system was utilized to generate stable cell lines with specific gene knockdown or overexpression. Cells (1.5 × 10^5^/well) were added to six-well plates 4 h before transfection. The sequence of ANKHD1-BP3 siRNAs (5′–3′) was GCGTCTGGAGGATATGTTAAT. Plasmid pSLenti-U6-shRNA-CMV-EGFP-F2A-Puro-WPRE was purchased from the Obio Company (Shanghai, China). Plasmid CMV-GFP-3FLAG-puro-HSPB1 was purchased from NOVOBIO (Shanghai, China).

### Cell counting kit (CCK)-8 and colony formation assays

A commercial cell counting kit (CCK)-8 (Sigma Chemical, St. Louis, MO, USA) assay was used to evaluate cell proliferation. Cells were seeded onto 96-well plates at a density of 5 × 10^3^ cells per well and cultured at 37 °C with 5% CO_2_. Absorbance was measured after an additional 3 h of incubation. A microplate reader (Thermo Fisher Scientific, Waltham, MA, USA) was used to detect the absorbance at a wavelength of 450 nm.

At 48 h from transfection, cells were plated in 6 cm cell culture dishes (1000 cells/dish) and incubated for 14 days. Cells were then stained for 20 min with crystal violet and the number of colonies (>50 cells) was determined.

### Migration and invasion assays

For migration assays, cells were cultured for 24 h without Matrigel matrix. In the upper chamber, cells were cultured in a serum-free medium, and the lower chamber was filled with a 10% FBS medium. Cell invasion assay was performed using 6.5 mm transwells with 8.0 µm pore polycarbonate membrane inserts coated with a 0.5 mg/ml Matrigel matrix (BD, Franklin Lakes, NJ, USA) placed in a 24-well plate (Corning, Corning, NY, USA). Cells were seeded onto 24-well plates at a density of 1 × 10^5^ cells per well and cultured at 37 °C with 5% CO_2_ for 48 h. Non-invading cells on the upper membrane surface were removed, and cells that passed through the filter were fixed with 4% paraformaldehyde for 15 min and stained for 10 min with hematoxylin at room temperature. The number of invading cells was counted in five randomly selected high-power fields (200× magnification) under an Olympus IX73 inverted microscope (Olympus Corporation, Tokyo, Japan). The data are representative of three individual wells.

### TUNEL assay

TUNEL assays were performed using an Elabscience^®^ Tunel cell apoptosis detection kit (Elabscience, Wuhan, China). Cell smears were fixed with 4% paraformaldehyde for 10 min and washed three times with PBS. Protease K (Elabsciece) and 0.1% Triton X-100 (Sangon Biotech) were added and the mixture was incubated for 5 min. After washing with PBS, the mixture was incubated with balance solution (Elabsciece) for 30 min, and then the prepared TdT working solution (Elabsciece) was added and the mixture was incubated for 60 min. DAPI (Beyotime) was used for staining in the dark.

### Subcutaneous tumor formation and peritoneal tumor metastasis assays

Fifteen BALB/C nude mice (Viton Lever, China; 4 weeks old, female, 10–12 g) were raised in a semi-barrier system with constant temperature and humidity, and their drinking water and feed were strictly sterilized. The study was approved by the Animals Ethics Committee of Tongji Hospital, affiliated with Tongji University School of Medicine. The mice were divided into three cell-based groups (five/group) as follows: Ishikawa, Ishikawa shRNA-ANKHD1-BP3, and Ishikawa NC. The cell density in each group was adjusted to 5 × 10^6^ cells/mL, and 0.2 mL was injected subcutaneously into the back of the nude mice. The continuous observation was conducted; the animals were euthanized after 4 weeks, and the tumor was measured.

Another 15 BALB/C nude mice (Viton Lever; 4 weeks old, female, 10–12 g) were divided into three cell-based groups (five/group) as follows: Ishikawa, shRNA-ANKHD1-BP3, and SPC. The cell density was 1 × 10^6^ cells/mL, and 0.2 mL was administered by direct intraperitoneal puncture into the abdominal cavity of the nude mice.

### mRNA library construction and sequencing

Total RNA from HEC-1b and HSPB1-HEC-1b cell lines was extracted using Trizol reagent (Invitrogen, CA, USA) following the manufacturer’s procedure. RNA quantity and purity were obtained with a Bioanalyzer 2100 and an RNA 6000 Nano LabChip Kit (Agilent, Santa Clara, CA, USA) with RIN number >7.0. Poly(A) RNA was obtained from total RNA (5 µg) using poly-T oligo-attached magnetic beads using two rounds of purification. Following purification, the mRNA was fragmented into small pieces using divalent cations under elevated temperatures. Then the cleaved RNA fragments were reverse-transcribed to create the final cDNA library in accordance with the protocol for the TruSeq RNA Sample Preparation v2 (Cat. RS-122-2001, RS-122-2002) (Illumina, San Diego, CA, USA). The average insert size for the paired-end libraries was 300 bp (±50 bp). Paired-end sequencing was performed on an Illumina Hiseq 4000 at LC Sciences (Illumina), following the vendor’s recommended protocol.

### Chromatin immunoprecipitation sequencing

We used the ChIp Assay Kit (Biyotime) to perform the ChIp Assay in accordance with the manufacturer’s instructions. Cells were cross-linked with 1% formaldehyde for 10 min at 37 °C and quenched with 125 mM glycine for 5 min. The DNA fragments with 200–1000 bp were prepared and then immunoprecipitated with Protein A + G Magnetic beads coupled with anti-HSPB1 (Cell Signaling Technology, Boston, MA, USA) antibodies. After reverse crosslinking, ChIP and input DNA fragments were end-repaired and A-tailed using the NEBNext End Repair/dA-Tailing Module (E7442, NEB) followed by adapter ligation with the NEBNext Ultra Ligation Module (E7445, NEB). The DNA library was amplified in 15 cycles and sequenced using the Illumina HiSeq (Illumina) with 2 × 150 pairs.

### Statistical analysis

Statistical analysis was performed using R version 3.5.1 (Institute for Statistics and Mathematics, Vienna, Austria; www.r-project.org) (packages: impute, UpSetR, ggplot2, rms, glmnet, preprocessCore, forestplot, survminer, survivalROC, and beeswarm). SPSS Version 22.0 (IBM, Armonk, NY, USA) was used for experimental analyses. Differences between two groups were assessed with Student’s *t*-test, while variance was used for three groups. *P* values were two-sided, and we defined *P* < 0.05 as statistically significant.

### Reporting summary

Further information on research design is available in the Nature Research Reporting Summary linked to this article.

## Supplementary information

Supplementary material

Reporting Summary Checklist

## Data Availability

The datasets generated and/or analysed during the current study are available in the Supplementary Material and TCGA program (https://portal.gdc.cancer.gov).

## Code availability

All DNA and RNA sequencing and array data were deposited in a community-endorsed public repository. The BioProject ID is PRJNA808440.
