# Supplementary material for: Alternative ANKHD1 transcript promotes proliferation and inhibits migration in uterine corpus endometrial carcinoma
Source: NPJ Genom Med. 2022 Sep 29;7:56. doi: 10.1038/s41525-022-00321-0 (PMC9519915; doi:10.1038/s41525-022-00321-0)
Supplement: Supplementary file 1 — Supplementary material [file 41525_2022_321_MOESM1_ESM.pdf]

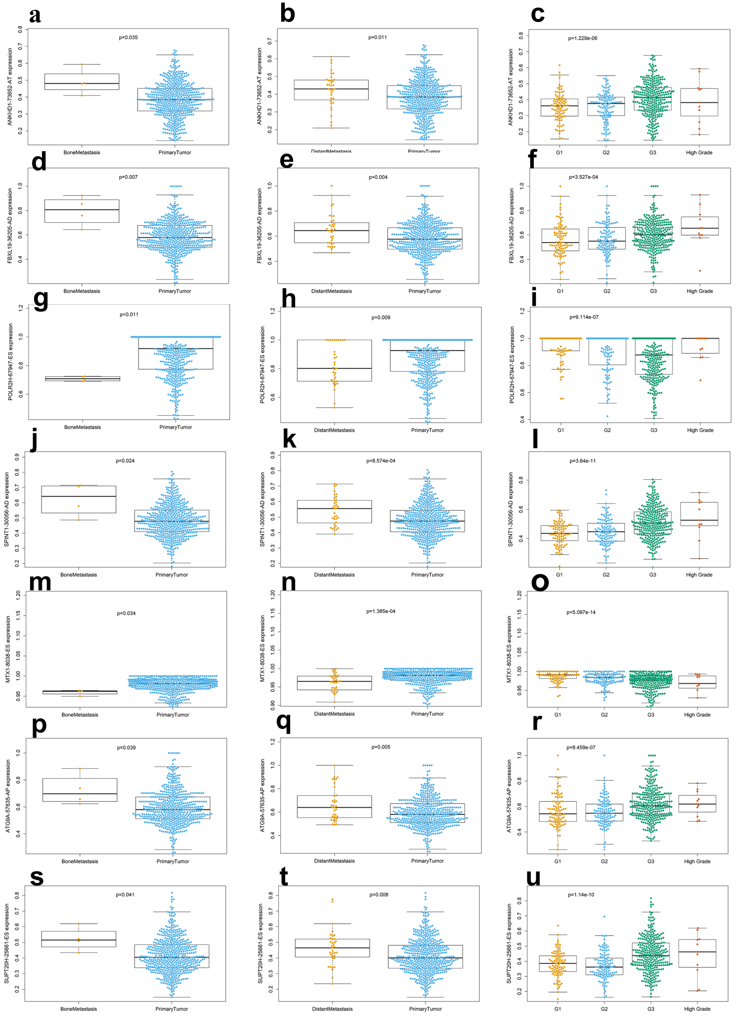

Supplementary Figure 1. The relationship between risk genes and clinical features. (a, d, g, j, m, p and s) bone metastasis; (b, e, h, k, n, q and t) distant metastasis and (c, f, i, l, o, r and u) tumor grades. Data shown are the mean±SD.

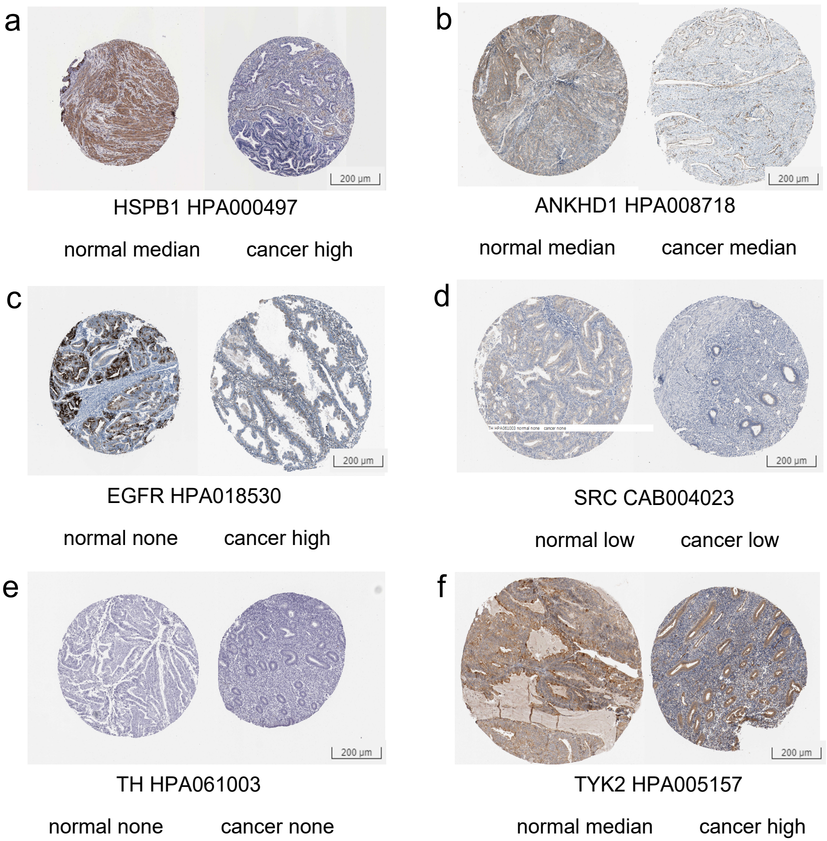

Supplementary Figure 2. Validation in the human protein atlas database.
The expression levels of HSPB1 (a), ANKHD1 (b), EGFR (c), SRC (d), TH (e) and TYK2 (f) in normal and tumor tissues by immunohistochemistry.

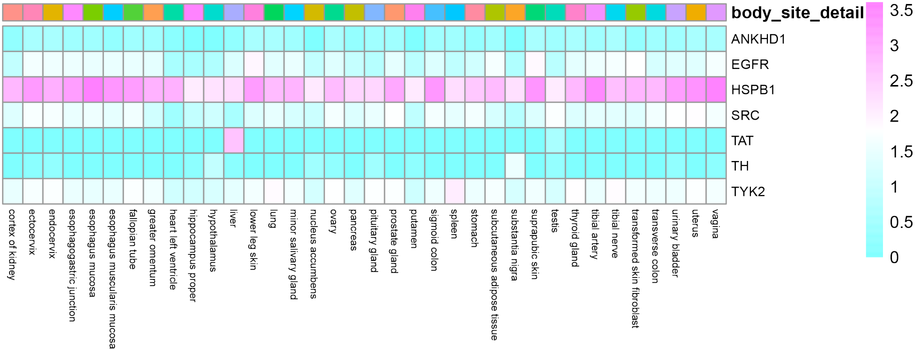

Supplementary Figure 3. Validation in the GTEx database.
The expfiles of HSPB1, ANKHD1, EGFR, SRC, TH, TYK2 and TAT.

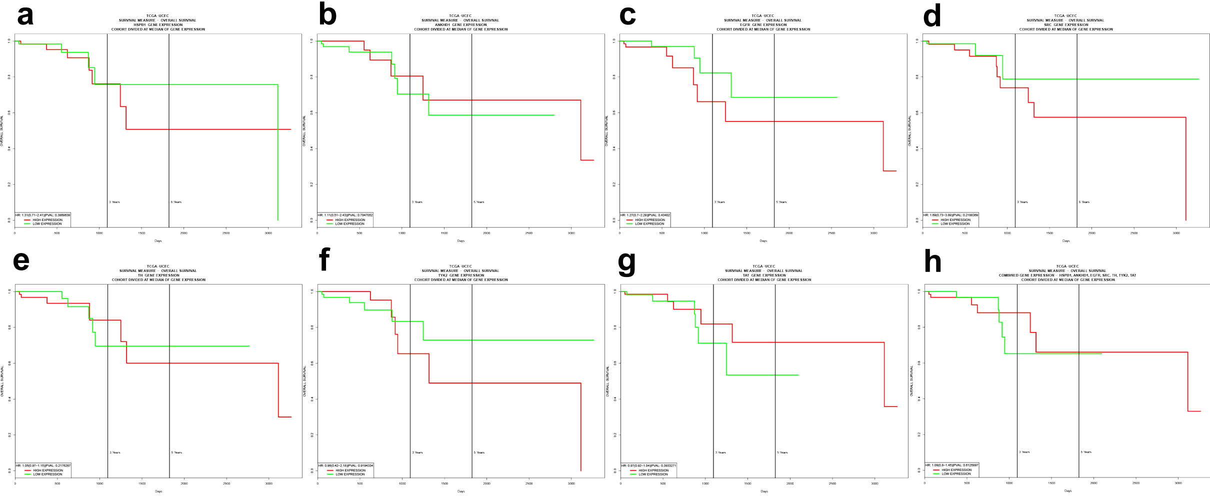

Supplementary Figure 4. Validation in the PROGgeneV2 database.
The overall survival curve of HSPB1 (a), ANKHD1 (b), EGFR (c), SRC (d), TH (e), TYK2 (f), TAT (g) and all genes integrated (h) in UCEC.

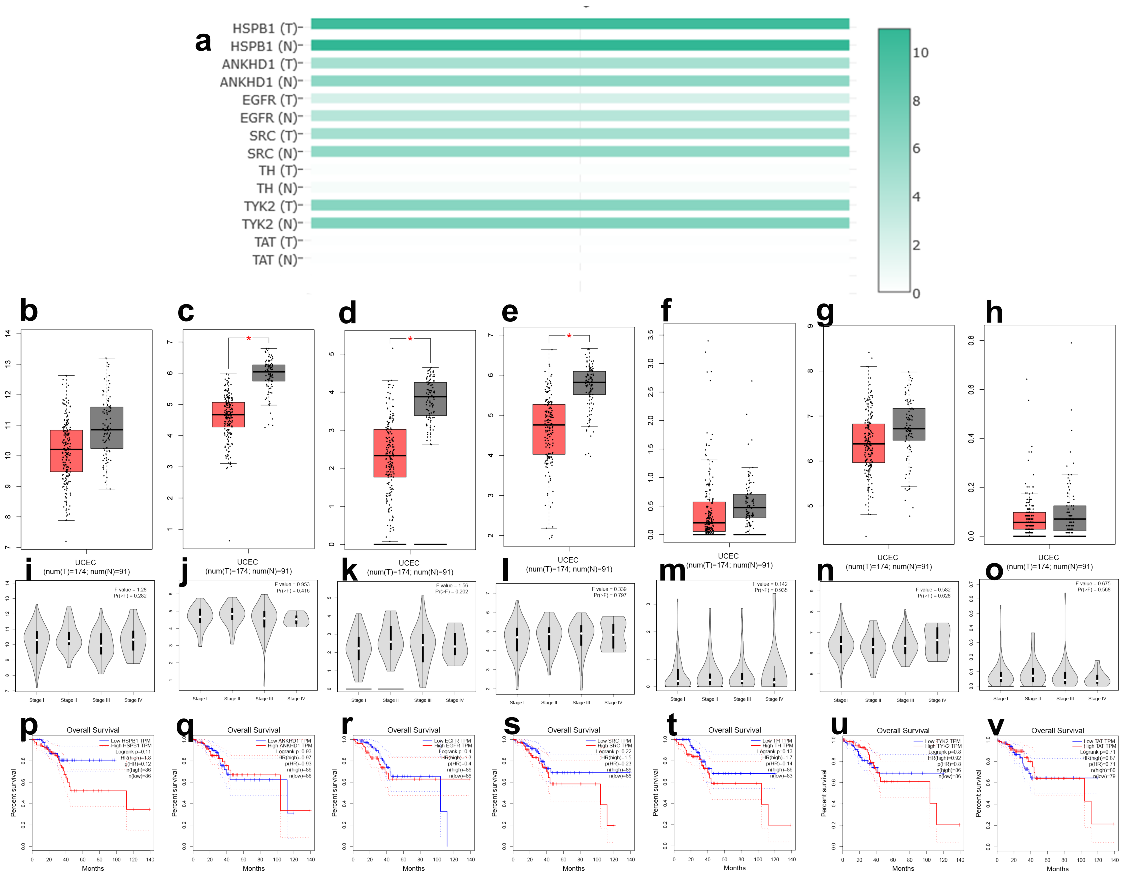

Supplementary Figure 5. Validation in the GEPIA database.

Different gene expressions in tumor and normal tissues (a-h). The association between gene expression levels and tumor stage (i-o); The association between gene expression levels and survival time (p-v). Data shown are the mean±SD

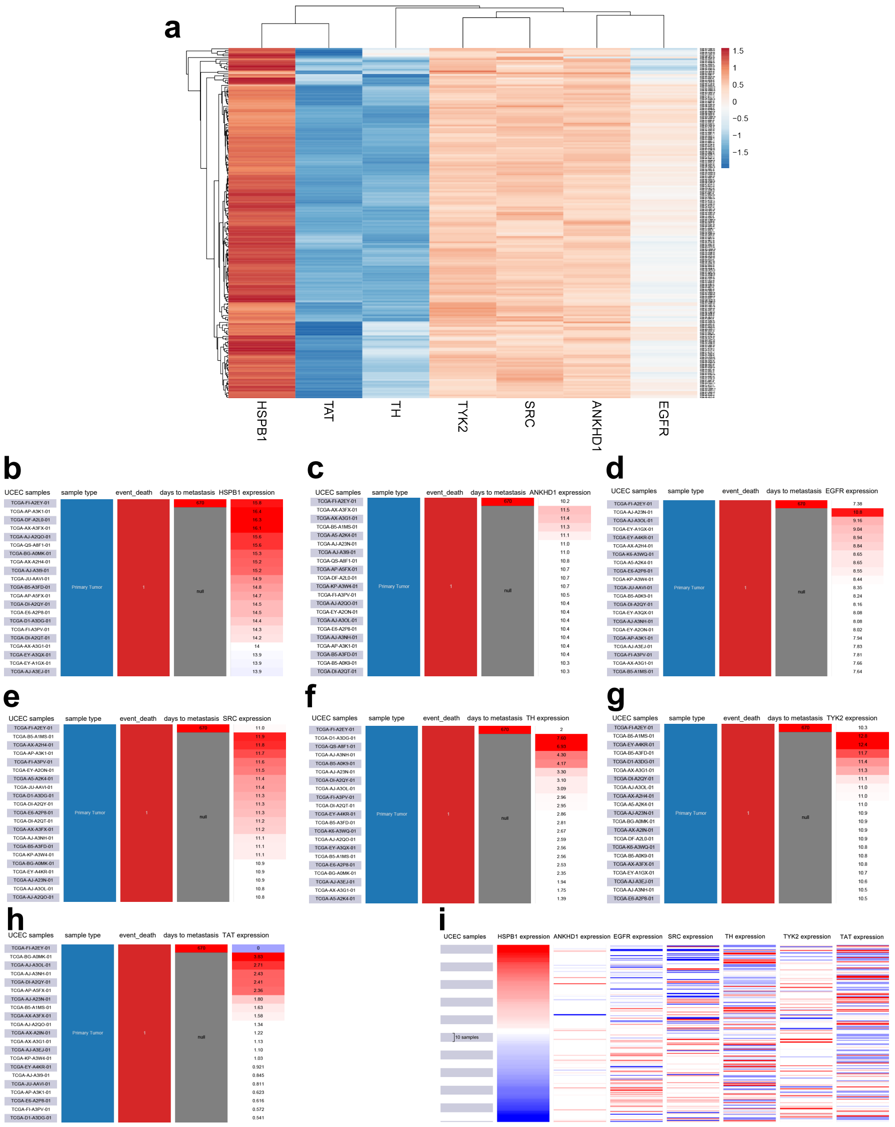

Supplementary Figure 6. Validation in the UCSC xena database.
Heatmap of genes in UCEC (a). The graph linked UCEC sample, primary type, metastasis and gene to show the relationship between genes and metastasis: HSPB1 (b), ANKHD1 (c), EGFR (d),SRC (e), TH (f), TYK2 (g), TAT (h) and all gene integrated (i).

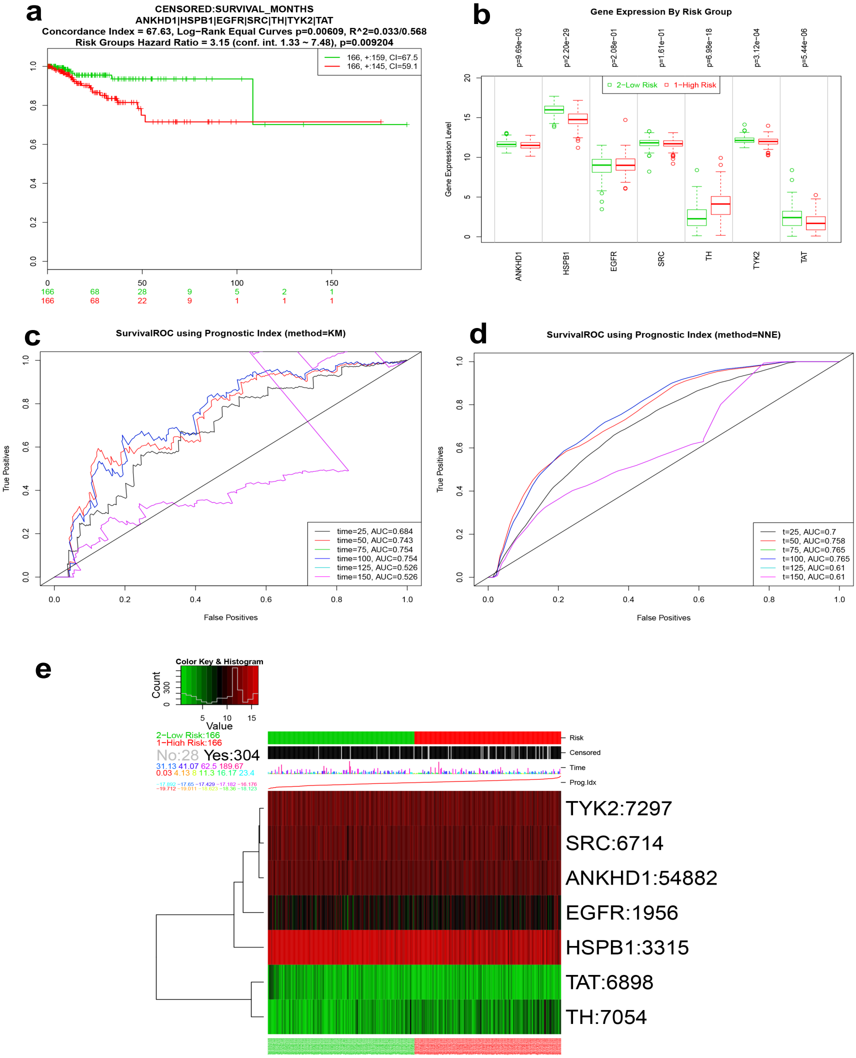

Supplementary Figure 7. Validation in the SurvExpress database.

Survival curve of integrated gene (a). Gene expression between high and low risk group (b). ROC curve of the integrated gene model (c, d). Expression heatmap of genes (e).

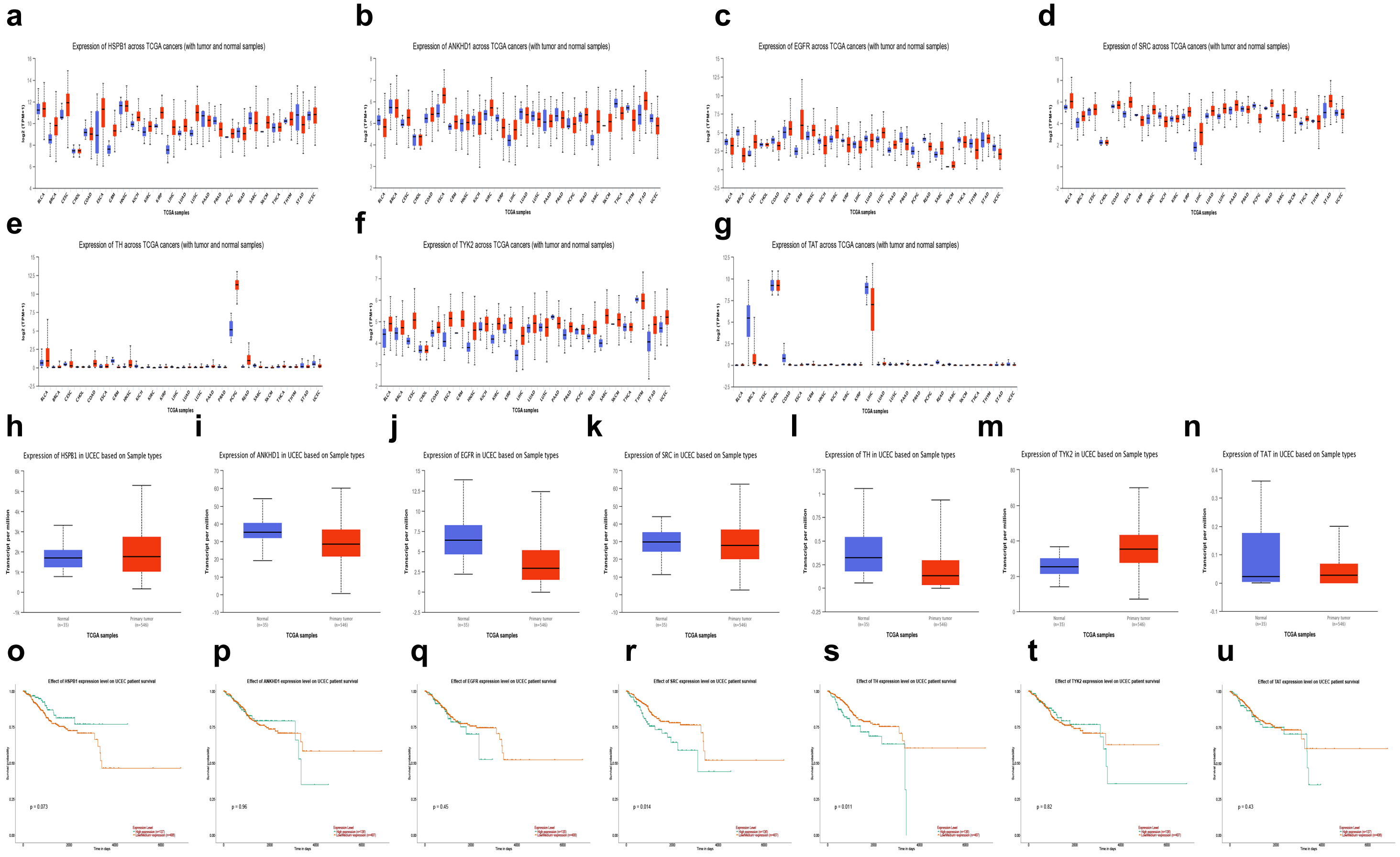

Supplement Figure 8. The validation in the UALCAN database.
Expression genes in TCGA cancer: HSPB1 (a), ANKHD1 (b), EGFR (c), SRC (d), TH (e), TYK2 (f) and TAT (g). Expression level based on sample types: HSPB1 (h), ANKHD1 (i), EGFR (j), SRC (k), TH (l), TYK2 (m) and TAT(n) in UCEC. Data shown are the mean±SD.

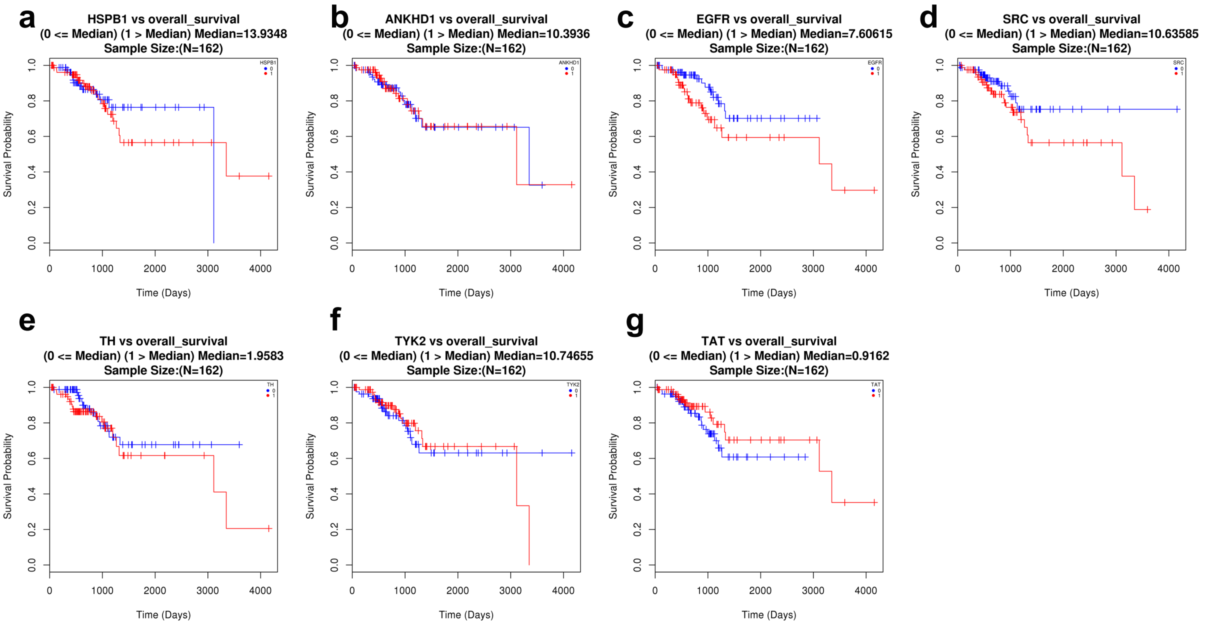

Supplementary Figure 9. Validation in the Linkedomics database.
Overall survival curve of HSPB1(a), ANKHD1 (b), EGFR (c), SRC (d), TH (e), TYK2 (f) and TAT (g) in UCEC.

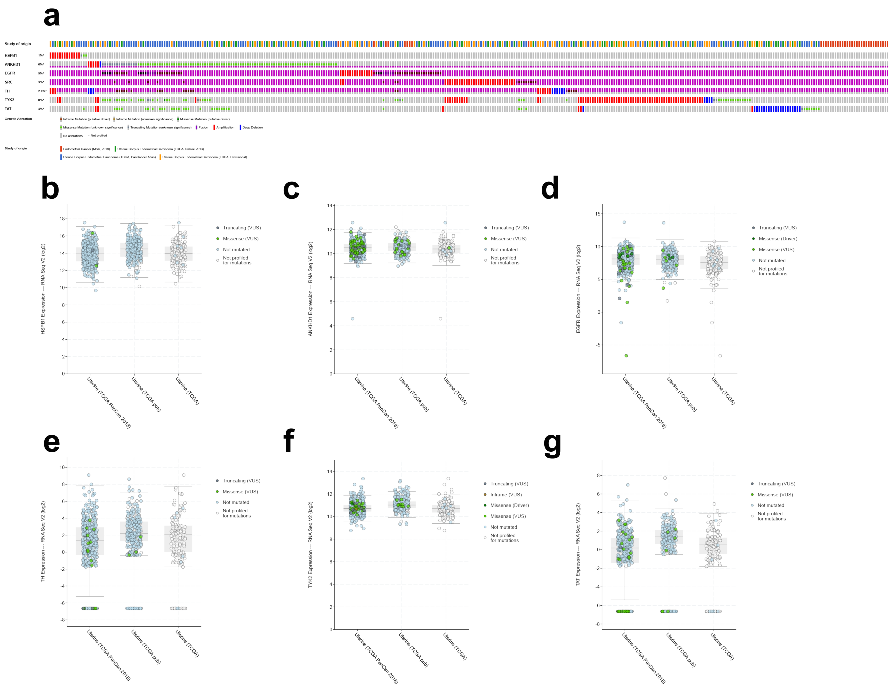

Supplementary Figure 10. Validation in the cBioportal database.
Integrated study of genes in cancer samples (a). Expression of HSPB1 (b), ANKHD1 (c), EGFR (d), TH (e), TYK2 (f) and TAT (g) in UCEC. Data shown are the mean±SD.

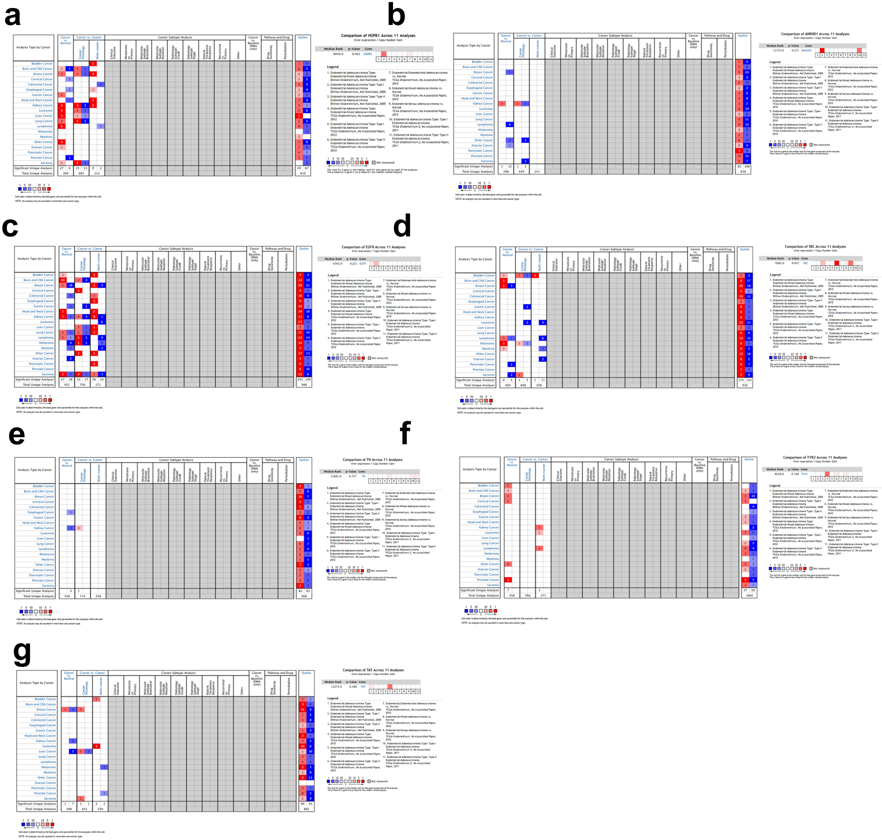

Supplementary Figure 11. Validation in the Oncomine database.
Expression of HSPB1 (a), ANKHD1 (b), EGFR (c), SRC (d), TH (e), TYK2 (f) and TAT (g) in 11 UCEC analysis.

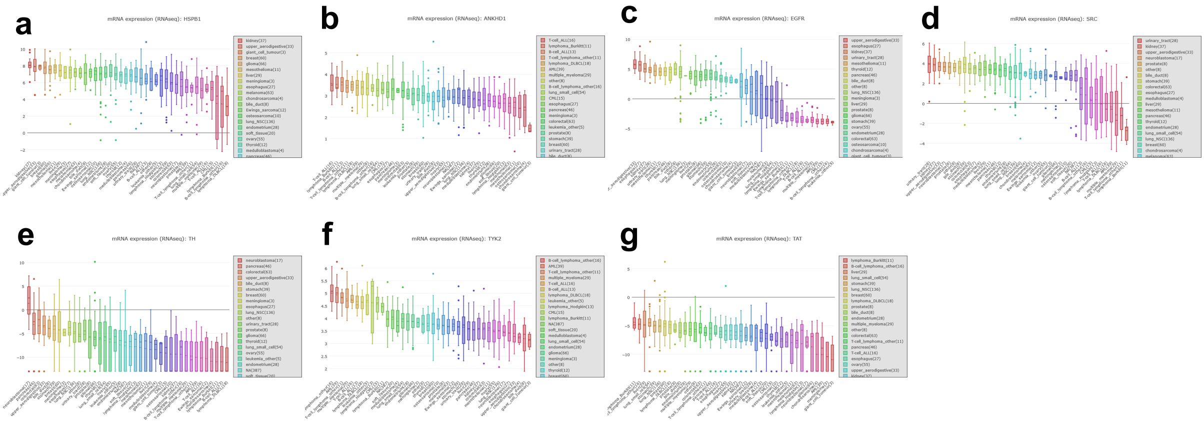

Supplementary Figure 12. Validation in the CCLE database.
Gene expression level in different cancer cell lines of HSPB1 (a), ANKHD1 (b), EGFR (c), SRC (d), TH (e), TYK2 (f) and TAT (g). Data shown are the mean±SD.

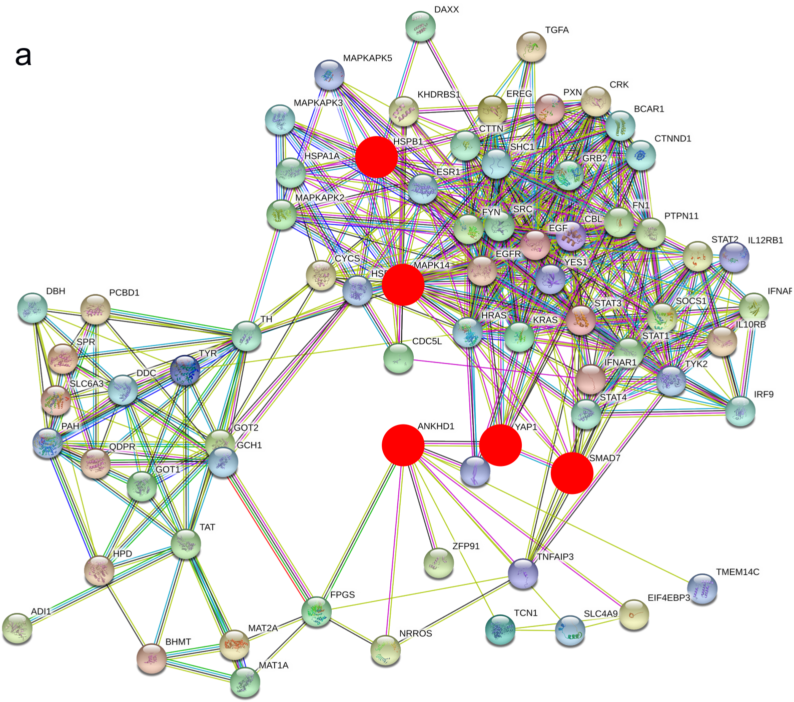

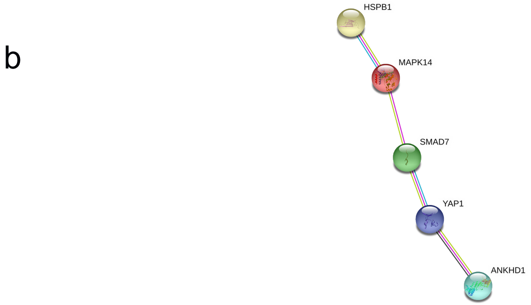

Supplementary Figure 13. Protein-Protein Interaction Networks (PPI).
Interaction network for HSPB1, ANKHD1, EGFR, SRC, TH, TYK2 and TAT (a). The simplify network of HSPB1 and ANKHD1 (b).

Supplementary Table 1. Mutual Exclusivity of HSPB1, ANKHD1, EGFR, SRC, TH, TYK2 and TAT in cBioportal database.

A	B	Neither	A Not B	B Not A	Both	Log2 Odds Ratio	p-Value	q-Value	Tendency
ANKHD1	EGFR	861	73	29	30	>3	<0.001	<0.001	Co-occurrence
ANKHD1	TAT	858	76	32	27	>3	<0.001	<0.001	Co-occurrence
EGFR	TYK2	856	34	78	25	>3	<0.001	<0.001	Co-occurrence
ANKHD1	TH	872	85	18	18	>3	<0.001	<0.001	Co-occurrence
ANKHD1	TYK2	818	72	72	31	2.29	<0.001	<0.001	Co-occurrence
SRC	TYK2	867	23	86	17	2.898	<0.001	<0.001	Co-occurrence
EGFR	TAT	890	44	44	15	2.786	<0.001	<0.001	Co-occurrence
EGFR	TH	909	48	25	11	>3	<0.001	<0.001	Co-occurrence
TH	TYK2	868	22	89	14	2.634	<0.001	<0.001	Co-occurrence
TYK2	TAT	847	87	43	16	1.857	<0.001	<0.001	Co-occurrence
TH	TAT	906	28	51	8	2.344	<0.001	0.001	Co-occurrence
SRC	TAT	902	32	51	8	2.145	0.002	0.003	Co-occurrence
EGFR	SRC	1089	60	34	7	1.902	0.006	0.01	Co-occurrence
HSPB1	TH	947	10	33	3	>3	0.01	0.015	Co-occurrence
SRC	TH	921	36	32	4	1.677	0.052	0.073	Co-occurrence
HSPB1	ANKHD1	877	13	103	0	<-3	0.239	0.295	Mutual exclusivity
HSPB1	TYK2	877	13	103	0	<-3	0.239	0.295	Mutual exclusivity
ANKHD1	SRC	855	98	35	5	0.318	0.402	0.469	Co-occurrence
HSPB1	EGFR	921	13	59	0	<-3	0.449	0.496	Mutual exclusivity
HSPB1	TAT	922	12	58	1	0.406	0.551	0.579	Co-occurrence
HSPB1	SRC	940	13	40	0	<-3	0.584	0.584	Mutual exclusivity

Supplementary Table 2. Summary of multidimensional external validation results base on multiple databases

	HSPB1		ANKHD1		EGFR		SRC		TH		TYK2		TAT		Results
	N	U	N	U	N	U	N	U	N	U	N	U	N	U	
**The human protein atlas**	-	↑	-	-	ND	↑	↓	↓	ND	ND	-	↑	NA	NA	HSPB1, EGFR and TYK2 high-expressed in tumor tissue (Figure S1).
**GTEx**	↑	NA	-	NA	-	NA	-	NA	-	NA	-	NA	-	NA	HSPB1 high-expressed in normal tissue (Figure S2).
**PROGgeneV2**	NA	↑	NA	↓	NA	↑	NA	↓	NA	-	NA	↑	NA	↓	HSPB1, EGFR and TYK2 high-expressed, but ANKHD1, TH and TAT low-expressed in UCEC in tissue level (Figure S3).
**GEPIA**	↑	↑	↑	↑	↑	↑	↑	↑	-	-	↑	↑	-	-	HSPB1, ANKHD1, EGFR, SRC and TYK2 high-expressed both in UCEC and normal in tissue level (Figure S4).
**UCSC xena**	NA	↑	NA	↑	NA	↑	NA	↑	NA	↑	NA	↑	NA	-	HSPB1, ANKHD1, EGFR, SRC, TH and TYK2 high-expressed in UCEC in tissue level (Figure S5).
**SurvExpress**	NA	↑	NA	↑	NA	↑	NA	↑	NA	-	NA	↑	NA	-	HSPB1, ANKHD1, EGFR and TYK2 high-expressed in UCEC in tissue level (Figure S6).
**UALCAN**	↑	↑	↑	↑	↑	-	↑	↑	-	-	↑	↑	-	-	HSPB1, ANKHD1, EGFR, SRC and TYK2 high-expressed, while HSPB1, SRC and TYK2 expressed higher in UCEC than in normal tissue level (Figure S7).
**Linkedomics**	NA	↑	NA	-	NA	↑	NA	↑	NA	↑	NA	-	NA	↓	HSPB1, EGFR, SRC and TH high-expressed, but TAT low-expressed in UCEC in tissue level (Figure S8).
**cBioportal**	NA	↑	NA	↑	NA	↑	NA	NA	NA	-	NA	↑	NA	-	HSPB1, ANKHD1, EGFR and TYK2 high-expressed in UCEC in tissue level (Figure S9).
**Expression atlas**	NA	↑	NA	↓	NA	-	NA	-	NA	↓	NA	-	NA	-	HSPB1 high-expressed, but ANKHD1 and TH low-expressed in UCEC in tissue level.
**Oncomine**	NA	↑	NA	↓	NA	↑	NA	-	NA	-	NA	↑	NA	-	HSPB1, EGFR and TYK2 high-expressed, but ANKHD1 low-expressed in UCEC in tissue level (Figure S10).
**CCLE**	NA	↑	NA	-	NA	↑	NA	↑	NA	↓	NA	↑	NA	↓	HSPB1, EGFR, SRC and TYK2 high-expressed, but TH and TAT low-expressed in UCEC in cellular level (Figure S11).

Note: "N" was defined as normal endarterium; "U" was defined as uterine corpus

endometrial carcinoma;“↑” was defined as a significantly high-expressed gene; “↓” was defined as a significantly low-expressed gene; “NA” was defined as “Not available”; “-” was defined as a gene with no significant difference in expression.

Supplementary Table 3. DNA library construction and sequencing

ID	Sequence
>BAND-1-ANKHD<br>1-BP3-Forward	NNNNNNNNNNNNNNNNNNATGCATCNNNATTATCAGACCCAAGCACATTCTCCCAACATCAGCCAAT<br>GGAGAGAGATGAT<br>TCTGGAATGGTAGCCCCCTCTAACATTTTTCATCAGCCTATGGCAAGTGGTTTTGTGGATTTTTCTAA<br>AGGTCTGCCAAT<br>TTCCATGTATGGAGGCACCATAATACCCTCTCATCCTCAGCTTGCTGATGTTCCAGGAGGCCCTCTG<br>TTTAATGGACTTC<br>ACAATCCAGATCCTGCTTGGAACCCTATGATAAAAGTTATCCAAAATTCAACTGAATGCACTGATGCC<br>CAGCAGGCCAGT<br>CTGCTTCCTTCAGTCCCTGCTCTCAAAGGGGAAATCCCATCACCTCAGCTAACCAGACCGAAGAAG<br>AGAATTGGACGGCC<br>GATGGTGGCCTCTCCTAACCAGAGGCACCNAGATCATCTACGACCGAAAGTTCCTNC
>BAND-1-ANKHD<br>1-BP3-Reverse	NNNNNNNNNNNNNNGGANAGGCCACCATCGGCCGTCCAATTCTCTTCTTCGGTCTGGTTAGCTGA<br>GGTGATGGGATTTCC<br>CCTTTGAGAGCAGGGACTGAAGGAAGCAGACTGGCCTGCTGGGCATCAGTGCATTCAGTTGAATTT<br>TGGATAACTTTTAT<br>CATAGGGTTCCAAGCAGGATCTGGATTGTGAAGTCCATTAAACAGAGGGCCTCCTGGAACATCAGC<br>AAGCTGAGGATGAG<br>AGGGTATTATGGTGCCTCCATACATGGAAATTGGCAGACCTTTAGAAAAATCCACAAAACCACTTGCC<br>ATAGGCTGATGA<br>AAAATGTTAGAGGGGGCTACCATTCCAGAATCATCTCTCTCCATTGGCTGATGTTGGGAGAATGTGC<br>TTGGGTCTGATAA<br>TTGTTGATGCATTGGATGGTTCCCCATCACAGATTGCGACCAACCTGATAAGCCTA

Figure 5c		
ANKHD1	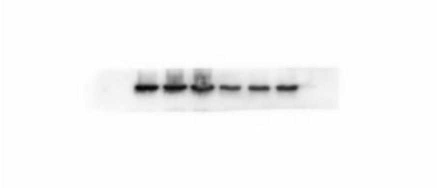	269KD
GAPDH	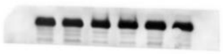	37KD
Figure 5f		
HEC-1-B		
ANKHD1		269KD
GAPDH	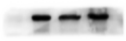	37KD
Ishikawa		

ANKHD1	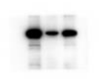	269KD
GAPDH	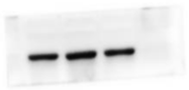	37KD
Figure 8b		
AKT	marker 55KD 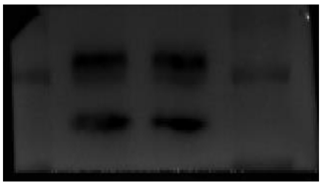 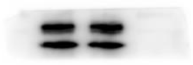	56KD
P-AKT	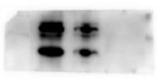	56KD
BCL-2	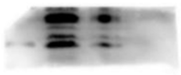	26KD

BAX	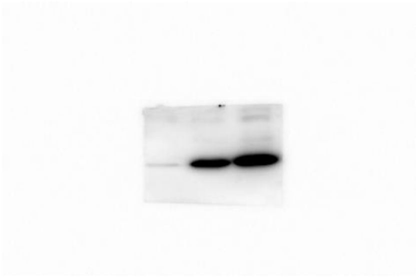	21KD
GAPDH	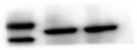	37KD
