# Supplementary material for: Alternative ANKHD1 transcript promotes proliferation and inhibits migration in uterine corpus endometrial carcinoma
Source: NPJ Genom Med. 2022 Sep 29;7:56. doi: 10.1038/s41525-022-00321-0 (PMC9519915; doi:10.1038/s41525-022-00321-0)
Supplement: Supplementary file 2 — Reporting Summary Checklist [file 41525_2022_321_MOESM2_ESM.pdf]

nature portfolio

Corresponding author(s): Yiqin Ouyang

Last updated by author(s): Jan 26, 2022

Reporting Summary

Nature Portfolio wishes to improve the reproducibility of the work that we publish. This form provides structure for consistency and transparency in reporting. For further information on Nature Portfolio policies, see our Editorial Policies and the Editorial Policy Checklist.

Statistics

For all statistical analyses, confirm that the following items are present in the figure legend, table legend, main text, or Methods section.

n/a	Confirmed	
☐	☒	The exact sample size (*n*) for each experimental group/condition, given as a discrete number and unit of measurement
☐	☒	A statement on whether measurements were taken from distinct samples or whether the same sample was measured repeatedly
☐	☒	The statistical test(s) used AND whether they are one- or two-sided<br>*Only common tests should be described solely by name; describe more complex techniques in the Methods section.*
☒	☐	A description of all covariates tested
☐	☒	A description of any assumptions or corrections, such as tests of normality and adjustment for multiple comparisons
☐	☒	A full description of the statistical parameters including central tendency (e.g. means) or other basic estimates (e.g. regression coefficient) AND variation (e.g. standard deviation) or associated estimates of uncertainty (e.g. confidence intervals)
☐	☒	For null hypothesis testing, the test statistic (e.g. *F*, *t*, *r*) with confidence intervals, effect sizes, degrees of freedom and *P* value noted<br>*Give P values as exact values whenever suitable.*
☒	☐	For Bayesian analysis, information on the choice of priors and Markov chain Monte Carlo settings
☒	☐	For hierarchical and complex designs, identification of the appropriate level for tests and full reporting of outcomes
☒	☐	Estimates of effect sizes (e.g. Cohen's *d*, Pearson's *r*), indicating how they were calculated

Our web collection on statistics for biologists contains articles on many of the points above.

Software and code

Policy information about availability of computer code

Data collection	Bioinformatics data were obtained from TCGA database.<br>RNAseq: We used endometrial cancer cells Ishikawa and HEC-1B cells, and overexpressed HSPB1 and transfected no-load plasmids as experimental group and control group, and observed the transcription and gene differences after overexpressed HSPB1. Endometrial cancer cells Ishikawa were used for down-knocking ANKHD1 and transfection of no-load plasmids. As the experimental group and control group, gene differences were observed after knocking down ANKHD1.<br>CHIPseq: HSPB1 was overexpressed in endometrial cancer cells Ishikawa and CHIP test was performed.
Data analysis	RNAseq: The original image data obtained by high-throughput sequencing is transformed into the original sequencing sequence by base recognition and analysis, which is called the original data, and the original data is stored in fastQ file format. Use Cutadapt to filter out disqualified sequences to get valid data. To obtain reads information, Hisat2 was used for reference genome comparison of Valid Data after pretreatment. Based on Hisat2 comparison results, Stringtie was used to reconstruct the transcript and calculate the expression levels of all genes in each sample. The volcano map can be used to understand the overall distribution of differentially expressed genes. Volcano maps were drawn for all genes in differential expression analysis using log2(FC) as abscissa and -log10(qvalue) as ordinate. Based on the results of gene difference analysis, GO enrichment analysis and KEGG enrichment analysis were performed. The results of KEGG enrichment analysis were presented in the form of bubble graph by GGploT2.<br>CHIP seq: The raw. Fastq files were trimmed by fastp software. The remained reads were mapped to the GRCh38 genome based on Bowtie2, a fast DNA aligner. MACS 2 was adopted for the identification of binding sites. ChIPseeker, a R bioconductor package, was used to annotate the peaks with their related genes, distance to the closest transcription start sites (TSS) and etc.

For manuscripts utilizing custom algorithms or software that are central to the research but not yet described in published literature, software must be made available to editors and reviewers. We strongly encourage code deposition in a community repository (e.g. GitHub). See the Nature Portfolio guidelines for submitting code & software for further information.

Data

Policy information about availability of data

All manuscripts must include a data availability statement. This statement should provide the following information, where applicable:

- Accession codes, unique identifiers, or web links for publicly available datasets
- A description of any restrictions on data availability
- For clinical datasets or third party data, please ensure that the statement adheres to our policy

Provide your data availability statement here.

Field-specific reporting

Please select the one below that is the best fit for your research. If you are not sure, read the appropriate sections before making your selection.

☒ Life sciences ☐ Behavioural & social sciences ☐ Ecological, evolutionary & environmental sciences

For a reference copy of the document with all sections, see nature.com/documents/nr-reporting-summary-flat.pdf

Life sciences study design

All studies must disclose on these points even when the disclosure is negative.

Sample size	In vivo, a total of 45 nude mice participated in the subcutaneous tumor formation experiment. The experiment was carried out with 15 nude mice at a time and divided into 3 groups with 5 mice in each group. Repeat 3 times. The same as abdominal metastasis experiment.
Data exclusions	No samples were excluded.
Replication	Repeat 3 times in vivo. More than 3 times in vitro.
Randomization	In vivo, subcutaneous tumor formation experiment and abdominal metastasis experiment, 15 nude mice were randomly assigned to 3 groups in each experiment.
Blinding	There was no double-blind both in vivo and vitro. After randomization, the operator conducts experimental treatment and needs to know the treatment factors of each group.

Reporting for specific materials, systems and methods

We require information from authors about some types of materials, experimental systems and methods used in many studies. Here, indicate whether each material, system or method listed is relevant to your study. If you are not sure if a list item applies to your research, read the appropriate section before selecting a response.

Materials & experimental systems

n/a	Involved in the study	
☐	☒	Antibodies
☐	☒	Eukaryotic cell lines
☒	☐	Palaeontology and archaeology
☐	☒	Animals and other organisms
☐	☒	Human research participants
☐	☒	Clinical data
☐	☐	Dual use research of concern

Methods

n/a	Involved in the study	
☐	☒	ChIP-seq
☐	☐	Flow cytometry
☐	☐	MRI-based neuroimaging

Antibodies

Antibodies used	Rabbit polyclonal to ANKHD1 antibody (Abcam, ab117788, 1:1000), goat anti-rabbit immunoglobulin G (Abcam, ab206718, 1:2000), Rabbit polyclonal anti-ANKHD1 (Bioss, bs-5831R, 1:100) , Rabbit monoclonal anti-GAPDH((Servicebio, GB11001, 1:1000), Bax (Abcam, ab182733, 1:2000), Bcl-2(Abcam, ab182858, 1:1000), Rabbit monoclonal Anti-Akt(Biotime, AF1777, 1:1000), Rabbit monoclonal Anti-Phospho-Akt(Biotime,AF1546, 1:1000), Rabbit monoclonal Anti-HSPB1(Cell Signaling Technology, 50353)
Validation	Rabbit polyclonal to ANKHD1 antibody, ab117788, species: human, validation: WB, IP. Rabbit polyclonal anti-ANKHD1, bs-5831R, species: human, Dog, Pig, Cow, Horse, Sheep, validation: WB, IHC, IF, ELISA. Rabbit monoclonal to Bax, ab182733, species: mouse, rat, human, validation: IP, WB. Rabbit monoclonal to Bcl-2, ab182858, species: mouse, human, validation: FC, WB, IHC-P. Rabbit monoclonal to Akt, AF1777, species: human, mouse, validaion: WB, IP, IF, IHC, ICC, FC. Rabbit monoclonal to Phospho-Akt, AF1546,

species: human, mouse, validaion: WB, IF, IHC, ICC. Rabbit monoclonal Anti-HSPB1, 50353, species: human, mouse, validation: WB, IP, IHC.

Eukaryotic cell lines

Policy information about cell lines

Cell line source(s)	Cells from human endometrial carcinoma, HEC-1-B bought from China Center for Type Culture Collection(CCTCC),GDC0129, Ishikawa bought from Huheng biology, FH0305.
Authentication	All cells have been tested by STR
Mycoplasma contamination	Negative
Commonly misidentified lines (See ICLAC register)	None.

Animals and other organisms

Policy information about studies involving animals; ARRIVE guidelines recommended for reporting animal research

Laboratory animals	Fifteen BALB/ C nude mice (Viton Lever, China), 4 weeks old, female, 10-12g, were raised in a semi-barrier system with constant temperature and humidity, and their drinking water and feed were strictly sterilized. The mice were divided into 3 groups (5/group): Ishikawa cell group, Ishikawa shRNA-ANKHD1-BP3 cell group, and Ishikawa NC cell group. Cell density in each group was adjusted to 5×106 cells /mL, and 0.2 mL was injected subcutaneously into the back of nude mice. Continuous observation was carried out, the animals were sacrificed after 4 weeks, and the tumor was measured.<br>Fifteen BALB/ C nude mice (Viton Lever, China), 4 weeks old, female, 10-12g were divided into 3 groups (5/group): direct intraperitoneal puncture inoculation, 0.2 mL of 1×106 Ishikawa, shRNA-ANKHD1-BP3 cell, and the SPC cell group were directly injected into the abdominal cavity of nude mice.
Wild animals	The stugy did not involve wild animals.
Field-collected samples	The study did not involve field-collected samples.
Ethics oversight	The present study was endorsed by the Ethics Committee of the Shanghai Tongji Hospital affiliated to Tongji University.

Note that full information on the approval of the study protocol must also be provided in the manuscript.

Human research participants

Policy information about studies involving human research participants

Population characteristics	A total of 36 female patients, including 15 metastatic UCEC tissues, 15 primary UCEC tissues, diagnosed with endometrial cancer, were collected between January 2019 and January 2020 at the Tongji Hospital affiliated to Tongji University School of Medicine.
Recruitment	We recruited patients who underwent endometrial cancer surgery in Tongji Hospital ffiliated to Tongji University School of Medicine in 2019-2020 without biases, and signed informed consent forms.
Ethics oversight	The present study was endorsed by the Ethics Committee of the Shanghai Tongji Hospital affiliated to Tongji University.

Note that full information on the approval of the study protocol must also be provided in the manuscript.

Clinical data

Policy information about clinical studies

All manuscripts should comply with the ICMJE guidelines for publication of clinical research and a completed CONSORT checklist must be included with all submissions.

Clinical trial registration	Do not apply
Study protocol	A total of 36 tumor specimens, including 15 metastatic UCEC tissues, 15 primary UCEC tissues, another 6 UCEC tissues, with paired non-tumor tissues, were collected between January 2019 and January 2020 at the Tongji Hospital affiliated to Tongji University School of Medicine. Written informed consent was obtained from all patients, and the procedures were approved by the Institutional Research Ethics Committee of Tongji Hospital affiliated to Tongji University School of Medicine. The 15 metastatic UCEC tissues and 15 primary UCEC tissues were used for immunohistochemistry to detect expression ANKHD1, and the 3 samples of UCEC tissues and paired non-tumor tissues were used for western blotting, and 6 for RT-PCR.
Data collection	A total of 36 tumor specimens, including 15 metastatic UCEC tissues, 15 primary UCEC tissues, another 6 UCEC tissues, with paired non-tumor tissues
Outcomes	Compared with metastasis UCEC samples, more ANKHD1 was detected in primary UCEC samples(Figure 5B) in six clinical samples and WB (Figure 5C) in three clinical samples. The expression levels showed significant difference, and ANKHD1 were expressed at low levels in carcinoma and at high levels in pare-carcinoma; protein levels were also high.

Dual use research of concern

Policy information about dual use research of concern

Hazards

Could the accidental, deliberate or reckless misuse of agents or technologies generated in the work, or the application of information presented in the manuscript, pose a threat to:

No	Yes	
☒	☐	Public health
☒	☐	National security
☒	☐	Crops and/or livestock
☒	☐	Ecosystems
☒	☐	Any other significant area

Experiments of concern

Does the work involve any of these experiments of concern:

No	Yes	
☒	☐	Demonstrate how to render a vaccine ineffective
☒	☐	Confer resistance to therapeutically useful antibiotics or antiviral agents
☒	☐	Enhance the virulence of a pathogen or render a nonpathogen virulent
☒	☐	Increase transmissibility of a pathogen
☒	☐	Alter the host range of a pathogen
☒	☐	Enable evasion of diagnostic/detection modalities
☒	☐	Enable the weaponization of a biological agent or toxin
☒	☐	Any other potentially harmful combination of experiments and agents

ChIP-seq

Data deposition

☒ Confirm that both raw and final processed data have been deposited in a public database such as GEO.

☒ Confirm that you have deposited or provided access to graph files (e.g. BED files) for the called peaks.

Data access links<br>*May remain private before publication.*	The sequence data have been uploaded to the public database, but we have not got the accession number yet. We will supplement the statement as soon as we get the accession number.
Files in database submission	Sampale name:Ishikawa HSPB1, organism: human species, isolate: protein, biomaterial provider:Wenhuizi Sun, sex: female, cell line: Ishikawa.
Genome browser session<br>(e.g. UCSC)	Do not suit.

Methodology

Replicates	1
Sequencing depth	The depth for Hsp is 3.62, Total reads is 72.38M, unique mapped reads is 54.84M, pair-end sequencing, read length is 2X150bp. The depth for Hspb1-input is 2.92, Total reads is 58.32M, unique mapped reads is 35.98M, pair-end sequencing, read length is 2X150bp.
Antibodies	HSP27 Rabbit mAb, #50353
Peak calling parameters	The software for peakcalling is MACS2, the parameter as follows: -f BAMPE --keep-dup auto -g 3099722973 -B -q 0.01
Data quality	5942 peaks are at FDR 0.01, 2934peaks are at 5-fold enrichment.
Software	The raw. Fastq files were trimmed by fastp software. The remained reads were mapped to the GRCh38 genome based on Bowtie2, a

Software	fast DNA aligner. MACS 2 was adopted for the identification of binding sites. ChIPseeker, a R bioconductor package, was used to annotate the peaks with their related genes, distance to the closest transcription start sites (TSS) and etc.

Flow Cytometry

Plots

Confirm that:

- [ ] The axis labels state the marker and fluorochrome used (e.g. CD4-FITC).
- [ ] The axis scales are clearly visible. Include numbers along axes only for bottom left plot of group (a 'group' is an analysis of identical markers).
- [ ] All plots are contour plots with outliers or pseudocolor plots.
- [ ] A numerical value for number of cells or percentage (with statistics) is provided.

Methodology

Sample preparation	Do not apply
Instrument	Do not apply
Software	Do not apply
Cell population abundance	Do not apply
Gating strategy	Do not apply

- [ ] Tick this box to confirm that a figure exemplifying the gating strategy is provided in the Supplementary Information.

Magnetic resonance imaging

Experimental design

Design type	Do not apply
Design specifications	Do not apply
Behavioral performance measures	Do not apply

Acquisition

Imaging type(s)	Do not apply
Field strength	Do not apply
Sequence & imaging parameters	Do not apply
Area of acquisition	Do not apply

Diffusion MRI ☐ Used ☒ Not used

Preprocessing

Preprocessing software	Do not apply
Normalization	Do not apply
Normalization template	Do not apply
Noise and artifact removal	Do not apply
Volume censoring	Do not apply

Statistical modeling & inference

Model type and settings	Do not apply
Effect(s) tested	Do not apply

Specify type of analysis: ☐ Whole brain ☐ ROI-based ☐ Both

Statistic type for inference (See Eklund et al. 2016)	Do not apply
Correction	Do not apply

Models & analysis

n/a	Involved in the study
☒	☐ Functional and/or effective connectivity
☒	☐ Graph analysis
☒	☐ Multivariate modeling or predictive analysis
